# Repurposing Amiodarone for Bladder Cancer Treatment

**DOI:** 10.1158/2767-9764.CRC-24-0433

**Published:** 2025-06-04

**Authors:** Francisco J. Roa, Maria Roubelakis, Konstantinos Paschidis, Nils C.H. van Creij, Florian Handle, Manousos Makridakis, Shaman Narayanasamy, Irina-Afrodita Balaur, Aggeliki Tserga, Antonia Vlahou, Frédéric R. Santer, Per-Sonne Holm, Michele Hoffmann, Martin Puhr, Marika Mokou, Maria Frantzi, Reinhard Schneider, Agnieszka Latosinska, Harald Mischak, Venkata Satagopam, Zoran Culig, Renate Pichler

**Affiliations:** 1 Department of Urology, Medical University of Innsbruck, Innsbruck, Austria.; 2 Laboratory of Biology, Medical School, National and Kapodistrian University of Athens, Athens, Greece.; 3 Biomedical Research Foundation, Academy of Athens, Athens, Greece.; 4 XPseq Analytics GmbH, Innsbruck, Austria.; 5 Luxembourg Centre For Systems Biomedicine (LCSB), University of Luxembourg, Esch-sur-Alzette, Luxembourg.; 6 Department of Oral and Maxilofacial Surgery, Medical University of Innsbruck, Innsbruck, Austria.; 7 Department of Urology, Medical Faculty and University Hospital Düsseldorf, Heinrich Heine University of Düsseldorf, Düsseldorf, Germany.; 8 Department of Biomarker Research, Mosaiques Diagnostics, Hannover, Germany.

## Abstract

Cisplatin-based neoadjuvant chemotherapy followed by radical cystectomy is the main treatment for muscle-invasive bladder cancer (MIBC). However, low survival rates highlight the necessity for new therapeutic strategies. Drug repurposing has emerged as a promising approach in cancer treatment, with various studies proposing the use of existing drugs for the treatment of bladder cancer. In this context, we previously established an *in silico* repurposing strategy using patient omics signatures, identifying drugs and compounds with the potential to reverse nonmuscle-invasive bladder cancer (NMIBC) to less aggressive subtypes. In the present study, we expanded our *in silico* approach to verify a list of compounds with potential antitumor activity against MIBC. We investigated the efficacy of the predicted candidates in a group of different bladder cancer cell lines, including NMIBC and MIBC. The most potent compound for decreasing cell viability was amiodarone, an antiarrhythmic drug widely used in the field of cardiology. Amiodarone reduced cell proliferation and colony formation capacity, with a stronger effect on the most aggressive invasive models, validating our repurposing pipeline. The drug additionally induced cell death and inhibited the activity of mTOR and its target protein S6, suggesting that the anticancer effect of the drug is, in part, mediated by inhibition of the mTOR signaling pathway. Furthermore, the administration of amiodarone in a xenograft MIBC mouse model reduced tumor growth without inducing toxicity. Altogether, we demonstrated that amiodarone is a potential repurposed drug for bladder cancer, which might be especially effective in MIBC.

**Significance:**

Treatment of advanced bladder cancer remains a therapeutic challenge in urological oncology. In order to make more drugs available to patients in the future, we identified amiodarone, a repurposed drug used in cardiology as a compound that inhibits bladder cancer *in vitro* and *in vivo*.

## Introduction

Bladder cancer is the ninth most prevalent cancer worldwide. For 2024, it is estimated that 83,190 new cases of bladder cancer will be diagnosed in the United States (about 63,070 in men and 20,120 in women). According to projections, 16,840 deaths (about 12,290 in men and 4,550 in women) could be expected (1, 2). Bladder cancer can be histologically classified as nonmuscle-invasive bladder cancer (NMIBC), muscle-invasive bladder cancer (MIBC), and metastatic disease (3). NMIBC represents approximately 75% of new cases, and diagnosed patients have a good life expectancy (4). Therapies for NMIBC include transurethral resection of the bladder tumor, adjuvant intravesical instillation therapies with Bacillus Calmette–Guérin (BCG), or chemotherapy (mitomycin C, epirubicin, and gemcitabine), depending on the European Association of Urology NMIBC risk groups (4–6). On the other hand, MIBC is currently treated with cisplatin-based neoadjuvant chemotherapy followed by radical cystectomy as a standard of care (4, 5). However, the pathological complete response to neoadjuvant chemotherapy treatment is limited, achieving pathological complete response rates of 36% to 42% when comparing gemcitabine/cisplatin with dose-dense methotrexate, vinblastine, doxorubicin, and cisplatin (7). Chemoresistance involves different molecular mechanisms, such as altered DNA damage repair (8). Although new combination therapies (chemotherapy plus immune checkpoint inhibition and immune checkpoint inhibition plus antibody–drug conjugates) in the perioperative setting are currently under evaluation in clinical trials (NCT03732677, NCT04960709, NCT03924895, and NCT04700124), optimized treatments to minimize the evolution of therapeutic resistance are still lacking (9).

Drug repurposing is the strategy of using approved drugs for new medical indications (10), representing a promising alternative to overcome the long and costly process of new drug discovery and development (11). This strategy has been widely studied in various types of cancer, and drugs with diverse indications are currently undergoing different phases of clinical trials to assess their anticancer effects (10). One of the most representative examples of drug repurposing is the BCG vaccine, which had been primarily used for the prevention of tuberculosis and was approved in 1990 by the FDA as an effective intravesical therapy for high-risk NMIBC to prevent recurrence and tumor progression (12). To date, BCG is still one of the few noncancer drugs that have been approved for cancer treatment by a health authority and is still the standard of care in the adjuvant setting of high-risk NMIBC, according to the European Association of Urology guidelines (13, 14). Similarly, several potential repurposed drugs for bladder cancer have been described at preclinical stages. For this purpose, compounds were selected based on drug screening (15–18) or previous observations suggesting their use in cancer treatment (19–23), and different molecular mechanisms explaining the effects of these drugs in bladder cancer have been proposed. Therefore, drug repurposing offers a promising opportunity to use derisked drugs to target specific molecular pathways implicated in bladder cancer pathogenesis, thus accelerating drug development.

We previously established a repurposing pipeline based on the integration of multiple molecular expression (-omics) data from hundreds of patients with bladder cancer (24, 25). This approach uses the Connectivity Map (CMap) resource, a well-known tool for data-driven drug repurposing (26, 27), to identify compounds with potential activity against bladder cancer. Using this strategy for high-risk NMIBC, we identified three mTOR inhibitors among the compounds with a high score for reversing aggressive bladder cancer molecular signatures. The compound that ranked first, WYE-354, decreased cell proliferation and colony growth in a panel of various bladder cancer cell lines without affecting apoptosis (24). This was the first repurposing strategy based on patients’ data that has been applied to bladder cancer and has successfully identified a compound with antiproliferative activity against bladder cancer *in vitro*.

In the present study, we expanded our previously published pipeline to integrate additional proteotranscriptomic signatures from patients with MIBC and NMIBC. This allowed us to obtain a list of noncancer drugs with potential antitumor activity for bladder cancer, with a focus on MIBC. We then investigated the values of the predicted candidates in different bladder cancer cell lines. The most potent compound for decreasing cell viability was the antiarrhythmic drug amiodarone, and we further demonstrated that it reduced bladder cancer growth *in vitro* and *in vivo*. Moreover, amiodarone inhibited the mTOR and p44/p42 signaling pathways, which are molecular targets with high relevance in bladder cancer and can partially explain the observed phenotype.

## Materials and Methods

### Molecular bladder cancer signature and CMap analysis

The analysis was based on previously generated proteomics data (28). One hundred and twelve raw data files (NMIBC: *n* = 58 pTa, *n* = 38 pT1; MIBC: *n* = 16 pT2+) generated from LC/MS-MS were analyzed using MaxQuant version 1.6.7.0 and Proteome Discoverer (version 2.4). Transcriptomics meta-analysis data from 495 patients with NMIBC (*n* = 229 pTa, *n* = 262 pT1, and *n* = 4 pTis) and 563 patients with MIBC (*n* = 371 pT2, *n* = 146 pT3, and *n* = 46 pT4) were retrieved from previously published studies (29). For both omics approaches, differential expression analysis (MIBC vs. NMIBC) was conducted using the Mann–Whitney test, followed by adjustment for multiple testing using the Benjamini–Hochberg (BH) method. Differentially abundant proteins (DAP) were defined as significant in at least one software analysis (MaxQuant or Proteome Discover; BH-adjusted *P* < 0.05) and were detected in more than 10% of samples (in at least one analysis). Differentially expressed genes (DEG; BH-adjusted *P* < 0.05) with AUC > 0.6 were considered for downstream analysis. Literature data were retrieved from the BcCluster database (30). The DAPs, DEGs, and features from BcCluster were compiled into a unique bladder cancer molecular signature by initially inspecting the consistency of directionality (i.e., upregulation/downregulation) of each feature among these different datasets. Features that demonstrated inconsistencies in directionality across the different datasets were excluded. Detailed information regarding the processing and analysis of individual omics data is presented in Supplementary File S1. Subsequently, the functional relationships between the multiomics molecular signature and known drug compounds were analyzed using the CMap tool (26), as described in detail in Supplementary File S1. The focus was placed on drugs and compounds that exhibited a significant negative enrichment (*P* value < 0.05). The most promising drug candidates were selected based on information available in various web-based tools/public databases (Open Targets, PubChem, Drugs@FDA, FDA-Approved Drugs, MeSH, Kyoto Encyclopedia of Genes and Genomes, and Drug Repurposing Hub) and literature.

### Cell culture

Four human bladder cancer cell lines representing different grades of urothelial cancer (31) were used in the present study. UMUC3 (basal mesenchymal phenotype) was obtained from Sigma-Aldrich, HT1197 (G4) from ATCC (CRL1473), and BFTC905 (G3) and RT112 (G2) from the German Collection of Microorganisms and Cell Cultures GmbH. UMUC3 cells were cultured in DMEM, HT1197 in minimum essential medium supplemented with 1% sodium pyruvate and nonessential amino acids, RT112 in RPMI 1640, and BFTC905 in high glucose DMEM. These media were supplemented with 10% FBS Supreme (PAN-Biotech), 1× GlutaMAX (Gibco, Thermo Fisher Scientific), and 1× penicillin/streptomycin (Lonza). For the human bladder epithelial cells, HBLAK cells were obtained from CELLnTEC and cultured in CnT-NX-E culture medium (CELLnTEC). The cells were purchased from the companies mentioned above and were not authenticated during the 10-month experimental period. All cell lines were routinely tested for *Mycoplasma* infection by MycoStrip Detection Kit (InvivoGen).

### Chemicals

Amiodarone hydrochloride, benzylpenicillin (penicillin G) potassium, primidone, propantheline bromide, and sulfanilamide were purchased from Sigma-Aldrich. Amodiaquine dihydrochloride, carteolol hydrochloride, iobenguane sulfate, methylergometrine maleate, and tubocurarine chloride were obtained from Merck. Amikacin hydrate, bupropion (amfebutamone) hydrochloride, captopril, cefoxitin sodium, demeclocycline hydrochloride, flavoxate hydrochloride, fluvoxamine maleate, guaifenesin, isradipine, latamoxef sodium, methocarbamol, metolazone, molindone hydrochloride, novobiocin sodium, oxybuprocaine hydrochloride, and tinidazole were purchased from Selleckchem. Cefotetan disodium was obtained from BIOZOL. Stock solutions (10 mmol/L) were prepared by dissolving the compounds in DMSO or distilled water according to the manufacturer’s instructions. The compounds were aliquoted and stored at −80°C until use.

### Viability assays

Cells were seeded in 96-well plates (4,000, 5,000, 3,000, and 10,000 cells/well for UMUC3, HT1197, BFTC905, and RT112, respectively) and treated with increasing concentrations (0.1–100 μmol/L) of each compound or the corresponding solvent (vehicle). Viability was analyzed after 96 hours using the CellTiter 96 AQueous One Solution Cell Proliferation Assay (Promega) according to the manufacturer’s instructions and measured on a Cytation 5 plate reader (BioTek). The experiments were run in triplicates.

### Proliferation assays

Real-time proliferation assays were performed using an Incucyte S3 system. Cells were seeded in 96-well plates (4,000, 5,000, 3,000, 10,000, and 3,000 cells/well for UMUC3, HT1197, BFTC905, RT112, and HBLAK, respectively) and treated with increasing concentrations of amiodarone (2.5–50 μmol/L) or DMSO (vehicle and control). Confluence was measured every 4 hours for 96 hours using the Incucyte Base Analysis Software with AI Confluence segmentation.

### Colony formation assays

Cells were seeded at a low density in six-well plates (1,000, 3,000, 5,000, and 2,000 cells/well for UMUC-3, HT1197, BFTC-905, and RT112, respectively) and treated with increasing concentrations of amiodarone (1–5 μmol/L) or DMSO. After incubation for 7 to 10 days, cells were fixed with 50% methanol in PBS and stained with 0.5% crystal violet. The plates were scanned using a Canon scanner, and the number of colonies was determined using ImageJ software.

### Caspase activation assays

Cells were seeded in 96-well plates (4,000, 5,000, 3,000, and 10,000 cells/well for UMUC3, HT1197, BFTC905, and RT112, respectively) and treated with amiodarone (25 and 50 μmol/L for UMUC3; 12.5 and 25 μmol/L for HT1197, BFTC905, and RT112) or DMSO for 48 or 72 hours. Apoptosis was evaluated using the Caspase-Glo 3/7 Assay (Promega) according to the manufacturer’s instructions. Apoptosis was normalized to cell viability, which was measured as previously described. Both methods were analyzed using a Cytation 5 plate reader (BioTek).

### *In vivo* experiments

NOD/SCID mice were purchased from The Jackson Laboratory (JAX Mice & Services) and housed and maintained at the Animal Facility of the Biomedical Research Foundation of the Academy of Athens. The procedures for the care and treatment of animals were performed according to the Association for Assessment and Accreditation of Laboratory Animal Care International (approval nos. 3373/03-07-2018 and 350028/20-04-22) and the recommendations of the Federation of European Laboratory Animal Science Associations and NIH. The present protocol was approved by the Department of Agriculture and Veterinary Service of the Prefecture of Athens (permit number: 812707/23).

Amiodarone hydrochloride was purchased from STADAPHARM GmbH and dissolved in sterile water. For tumor formation, 4 × 10^6^ BFTC905 cells resuspended in 200 μL of PBS were administered subcutaneously into the tail base of 8- to 10-week-old NOD/SCID mice. Amiodarone treatment was initiated on the third day after injection by oral gavage of an aqueous solution and administered daily for 3 weeks. Two groups of mice were used: control (*n* = 6) and 75 mg/kg amiodarone (*n* = 6). To monitor tumor formation *in vivo*, tumor size was measured using calipers on a weekly basis for at least 27 days or until the presence of a tumor diameter >17 mm, tumor ulceration, or bleeding. In these cases, mice were sacrificed. The tumor volume was calculated using the modified ellipsoidal formula: V = ½ (length × width^2^). Animals were euthanized, and the tumors, liver, heart, and kidneys were collected.

### Histochemical analysis of tissue sections

The liver, heart, and kidney tissues were fixed in 10% formalin (Sigma-Aldrich) for 24 hours. Subsequently, they were washed with tap water for 10 minutes and placed in 70% ethanol. The tissues were then embedded in paraffin, and five- to seven-micron sections were prepared. Tissue sections were dewaxed in xylene (CARLO ERBA Reagents) and rehydrated in graded ethanol. Harris hematoxylin and 1% eosin (VWR) were used for morphological assessments. Hematoxylin and eosin–stained sections were washed with water and dehydrated with graded alcohol and xylene. Images were obtained using a bright-light microscope (Leica DM LS2 microscope and Leica DFC500 digital color camera).

### RNA sequencing

Total RNA was isolated from frozen xenograft tumor samples using the Quick-RNA MagBead kit (Zymo Research) in conjunction with ZR BashingBead Lysis Tubes (2.0 m; Zymo Research) according to the manufacturer’s protocol. Gene expression analysis was performed using the ExpressoSeq rapid 3′RNA-Seq service (XPseq Analytics GmbH). Raw sequencing reads were aligned to the human (GENCODE Release 47, GRCh38.p14) and mouse (GENCODE Release M36, GRCm39) reference genomes using the Minimap2 aligner (version 2.28). To remove reads originating from the mouse host, the R package XenofiteR (version 1.6) was employed. Read counting and differential gene expression analysis were performed with the Rsubread (version 2.18.0) and edgeR (version 4.2.2) packages in R (version 4.4.2). Gene set enrichment analysis was performed using the CAMERA function with the MSigDB Hallmark gene sets (version 2024. 1.Hs). Gene set activity scores were calculated with the Gene Set Variation Analysis package (1.52.3).

### Western blot analysis

Cells were lysed in RIPA buffer supplemented with protease (Merck) and phosphatase inhibitors (Sigma-Aldrich), and protein concentration was measured using the Bradford method. A total of 30 μg of protein was separated on NuPAGE Novex 4–12% Bis-Tris protein gels (Thermo Fisher Scientific) and transferred to 0.2 μm nitrocellulose membranes (GE HealthCare). The membranes were blocked for 1 hour with the StartingBlock Blocking Buffer (Thermo Fisher Scientific) and incubated overnight at 4°C with primary antibodies. Phospho-Akt (Ser473; #4060), Akt (#9272), phospho-mTOR (p-mTOR; Ser2448; #5536), mTOR (#2983), phospho-S6 ribosomal protein (p-S6; Ser235/236; #4858), S6 ribosomal protein (#2317), phospho-p44/42 MAPK (Erk1/2; Thr202/Tyr204; #4370), p44/42 MAPK (Erk1/2; #4695), and cleaved PARP (cPARP; #5625) were obtained from Cell Signaling Technology. GAPDH MAB374 (Millipore) and GAPDH ABS16 were obtained from Merck. The membranes were incubated for 45 minutes with IRDye secondary antibodies (LI-COR Biosciences) and scanned using an Odyssey CLx System (LI-COR Biosciences). The Image Studio software was used for quantification.

### Statistical analysis

GraphPad Prism 9 software was used for statistical analyses. Results are presented as the mean ± SEM. One-way ANOVA with Dunnett’s multiple comparisons test was used for multiple comparisons. The Student *t* test was used for comparing control and treated groups in the *in vivo* experiments. Significant differences are noted in the figures as *, *P* < 0.05; **, *P* < 0.01; ***, *P* < 0.001; and ****, *P* < 0.0001.

### Data availability

The raw sequencing data from this study have been deposited in the European Nucleotide Archive under accession number PRJEB88990 and are available for download at https://www.ebi.ac.uk/ena/browser/view/PRJEB88990. The read counts, log_2_ counts per million, and differential gene expression results are available in Supplementary File S2.

## Results

### Identification of drugs with antitumor activity in bladder cancer by *in silico* repurposing

**Figure 1:**
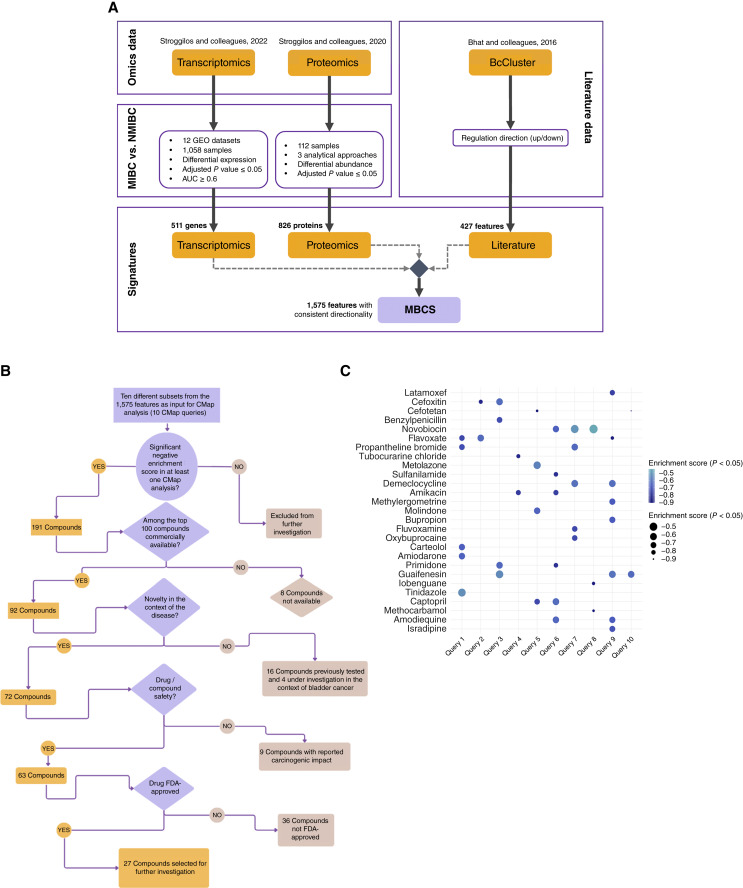
Development of molecular signature and CMap analysis. **A,** A molecular signature was established through the integration of significant changes between MIBC and NMIBC derived from multiomics data (proteomics and transcriptomics), further enriched with literature-mined data. **B,** The signature was randomly divided into 10 sets of molecular features and used to query the CMap to predict drugs/compounds affecting the disease’s molecular signature. **C,** The significant negative enrichment scores for the drugs selected for subsequent *in vitro* investigations are depicted. GEO, Gene Expression Omnibus; MIBC, muscle-invasive bladder cancer; NMIBC, non muscle-invasive bladder cancer.

To identify existing drugs that can be repurposed for MIBC, we expanded our previously published pipeline by including additional proteotranscriptomic signatures from patients with MIBC and NMIBC, utilizing them as input for *in silico* drug mapping (CMap) analysis. For the development of patients’ signatures, previously generated proteomics and transcriptomics data were reanalyzed. In particular, based on integrative transcriptomic analysis, we identified a total of 1,138 genes that were significantly differentially expressed between MIBC and NMIBC based on the Bonferroni-adjusted *P* value (*P* < 0.05), with the log fold change between MIBC and NMIBC ranging between −0.89 and 0.74. Among these 1,138 genes, 511 genes had an AUC ≥0.6 and were defined for the purpose of this study as DEGs, of which 295 were upregulated and 216 downregulated in MIBC versus NMIBC, respectively (Supplementary Table S1). Using proteomics, 870 proteins were found to be significantly altered in at least one of the mass spectrometry data analysis platforms (BH-adjusted *P* value <0.05), were detected in >10% of samples in at least 1 comparison, and showed agreement in fold change directionality among significant findings. Subsequently, 44 plasma-derived proteins were assumed to be contaminants and were excluded, resulting in a final list of 826 DAPs retained for downstream analyses, including 519 proteins with increased and 307 proteins with decreased abundance in MIBC compared with NMIBC (Supplementary Table S2). A combined bladder cancer molecular signature was generated by consolidating all features that were statistically significant in any of the omics analyses (i.e., proteomics/transcriptomics) or literature (BcCluster). Upon excluding features that exhibited contradictory regulation between different omics and/or literature data, we generated a molecular signature comprising 1,575 features that were either consistent or unique across the three independent resources (Fig. 1A).

CMap was used to identify information on inverse relationships between drugs and MIBC (as reflected in the signature of 1,575 features) characterized by negative enrichment. In total, 191 compounds were identified as having the potential to reverse the disease signatures. Among the top hits were compounds with known effects on bladder cancer, which further validated the approach. A total of 27 compounds with potential antitumor activity against bladder cancer were shortlisted. The selection criteria included the availability of the compound, novelty (no prior association with bladder cancer), absence of described potential carcinogenic effects, and FDA approval for other indications (Fig. 1B). These compounds included antibacterial agents (latamoxef, cefoxitin, cefotetan, benzylpenicillin, novobiocin, demeclocycline, and amikacin), acetylcholine receptor antagonists (flavoxate, propantheline bromide, and tubocurarine chloride), carbonic anhydrase inhibitors (metolazone and sulfanilamide), dopamine/serotonin receptor antagonists (methylergometrine, molindone, bupropion, and fluvoxamine), adrenergic receptor antagonists, potassium channel blockers (carteolol and amiodarone), and others (primidone, guaifenesin, iobenguane, tinidazole, captopril, methocarbamol, amodiaquine, isradipine, and oxybuprocaine; Fig. 1C).

### Amiodarone decreases cell proliferation and colony formation capacity in bladder cancer cell lines

The 27 drugs identified by the *in silico* repurposing algorithm were tested for efficacy in viability assays in a group of cell lines representing different bladder cancer stages and grades, that is, the noninvasive cell line RT112 and invasive cell lines UMUC3, HT1197, and BFTC905. Four of the 27 drugs tested (amiodarone, amodiaquine, fluvoxamine, and isradipine) decreased cell viability in the micromolar range (Supplementary Fig. S1). Amiodarone, an antiarrhythmic drug, had the most potent effect in the four cell models, as revealed by the percentage of decrease in viability and IC_50_ values (Supplementary Fig. S1). Based on these results, amiodarone was selected as a candidate for further analyses.

**Figure 2:**
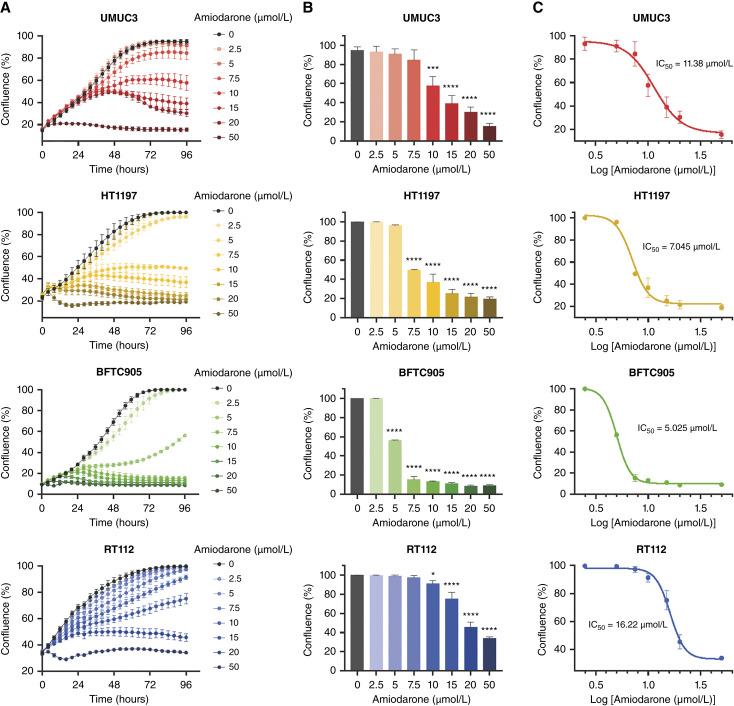
Amiodarone decreases proliferation in bladder cancer cell lines. Real-time proliferation assays in UMUC3, HT1197, BFTC905, and RT112 were conducted using the Incucyte S3 system. The cells were treated with increasing concentrations of amiodarone (0–50 μmol/L), and the confluence was measured every 4 hours over 96 hours. **A,** Cell confluence over time. **B,** Cell confluence after 96 hours of treatment. Data represent mean ± SEM from at least three independent experiments (one-way ANOVA with Dunnett’s multiple comparisons test; *, *P* < 0.05; ***, *P* < 0.001; ****, *P* < 0.0001). **C,** Concentration–response curves and IC_50_ values for amiodarone after 96 hours of treatment.

Real-time proliferation assays using the Incucyte system confirmed the anticancer effect of amiodarone, which reduced cell growth over time in a dose-dependent manner in the four cell lines (Fig. 2A). Amiodarone showed a stronger effect on BFTC905 and HT1197 cells, with a significant reduction in proliferation starting at 5 and 7.5 μmol/L, respectively (Fig. 2B). On the other hand, the noninvasive cell line RT112 was the most resistant to the treatment, as indicated by the IC_50_ values obtained for amiodarone: 5.025 μmol/L in BFTC905, 7.045 μmol/L in HT1197, 11.38 μmol/L in UMUC3, and 16.22 μmol/L in RT112 (Fig. 2C). The IC_50_ value of 3.9 μmol/L was reported for the receptor tyrosine kinase inhibitor erlotinib in the bladder cancer cell line T24 (32). Interestingly, treatment of the benign bladder epithelial cell HBLAK with amiodarone concentrations up to 20 μmol/L did not result in an antiproliferative effect (Supplementary Fig. S2A and S2B). The high resistance of HBLAK cells to amiodarone is reflected in the fact that no IC_50_ value could be determined, and it was reported as “ambiguous” in the concentration–response curve analysis (Supplementary Fig. S2C).

**Figure 3:**
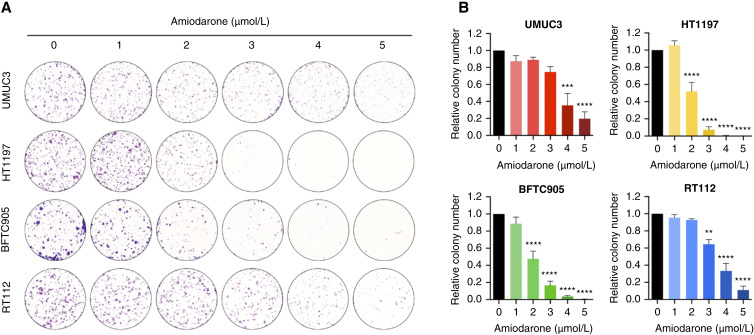
Amiodarone decreases colony formation capacity in bladder cancer cell lines. Cells seeded in six-well plates were treated with increasing concentrations of amiodarone (0–5 μmol/L). After incubation for 7 to 10 days, the cells were stained with crystal violet, and the plates were scanned. **A,** Representative images of the colonies. **B,** Quantification of the colony number. Data are presented as the number of colonies related to the control and represent mean ± SEM from three independent experiments (one-way ANOVA with Dunnett’s multiple comparisons test; **, *P* < 0.01; ***, *P* < 0.001; ****, *P* < 0.0001).

Furthermore, the effect of amiodarone on colony formation capacity was evaluated in the four cancer cell lines using low concentrations of the drug (1–5 μmol/L). The number of colonies was dose-dependently reduced by increasing the concentrations of amiodarone in all cell models, and in line with the proliferation results, a greater effect on BFTC905 and HT1197 was observed (Fig. 3A). A concentration of 3 μmol/L amiodarone strongly reduced the number of colonies by 85% to 90% in these two cell lines, whereas RT112 cells were again more resistant to the treatment. UMUC3 exhibited a response similar to that of amiodarone in RT112 (Fig. 3B).

Our results demonstrated that amiodarone decreased the proliferation and colony formation capacity of bladder cancer cell lines in a dose-dependent manner. The effect of amiodarone was stronger in the invasive models than in the noninvasive cells RT112, which validates our *in silico* approach for predicting candidates with anticancer activity specifically for MIBC. In addition, the proliferation results in the benign HBLAK cells indicate that the effect of amiodarone is bladder cancer cell–specific.

### Amiodarone induces cell death in bladder cancer cell lines

**Figure 4:**
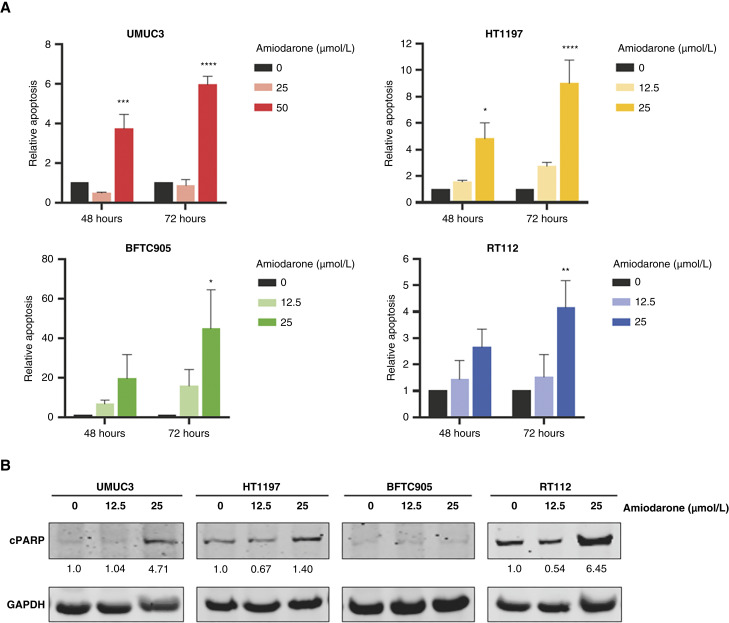
Amiodarone induces apoptosis in bladder cancer cell lines. Cells were treated for 48 or 72 hours with amiodarone (0, 25, and 50 μmol/L for UMUC3; 0, 12.5, and 25 μmol/L for HT1197, BFTC905, and RT112). **A,** Apoptosis was evaluated using the Caspase-Glo 3/7 Assay and viability using the CellTiter cell proliferation assay. Apoptosis results were normalized to viability. Data are presented as apoptosis related to the control and represent mean ± SEM from at least three independent experiments (one-way ANOVA with Dunnett’s multiple comparisons test; *, *P* < 0.05; **, *P* < 0.01; ***, *P* < 0.001; ****, *P* < 0.0001). **B,** Western blot analysis of cPARP expression after 48 hours of treatment with 0, 12.5, and 25 μmol/L amiodarone. GAPDH was used as a loading control. Blots are representative of three independent experiments. Quantification of cPARP expression (normalized by GAPDH) is indicated under each corresponding band. Values are presented as expression related to the control.

To elucidate whether the effect of amiodarone on bladder cancer cells was related to cell death, we performed apoptosis assays in our cell models. Amiodarone induced the activity of executioner caspases 3/7 at both 48 and 72 hours in the four cell lines, with a differential response for each model. Treatment with 25 μmol/L amiodarone for 48 hours increased caspase activity by 19.4-fold in BFTC905 cells, 4.8-fold in HT1197 cells, and 2.7-fold in RT112 cells compared with control cells (Fig. 4A). This difference was only statistically significant for HT1197 although BFTC905 showed higher caspase activation. A greater increase was observed at 72 hours, at which time caspase activity increased 44.7-fold in BFTC905, 9.0-fold in HT1197, and 4.2-fold in RT112 (Fig. 4A). There were no significant changes in caspase activity in UMUC3 cells treated with 25 μmol/L amiodarone; however, an increase of 3.7-fold at 48 hours and 5.9-fold at 72 hours was observed when the cells were treated with 50 μmol/L amiodarone (Fig. 4A).

In addition, Western blot assays were performed to detect the apoptosis marker cPARP. Treatment with 25 μmol/L amiodarone induced cPARP expression in UMUC3, HT1197, and RT112 cells, whereas no detection was observed in BFTC905 cells (Fig. 4B). Interestingly, BFTC905 was the cell line with the highest increase in caspase activity, which highlights the importance of using two different methods for detecting apoptosis.

Considering the combination of both methods used, our results showed that amiodarone induces cell death in bladder cancer cell lines.

### Amiodarone reduces tumor growth in a xenograft bladder cancer mouse model

**Figure 5:**
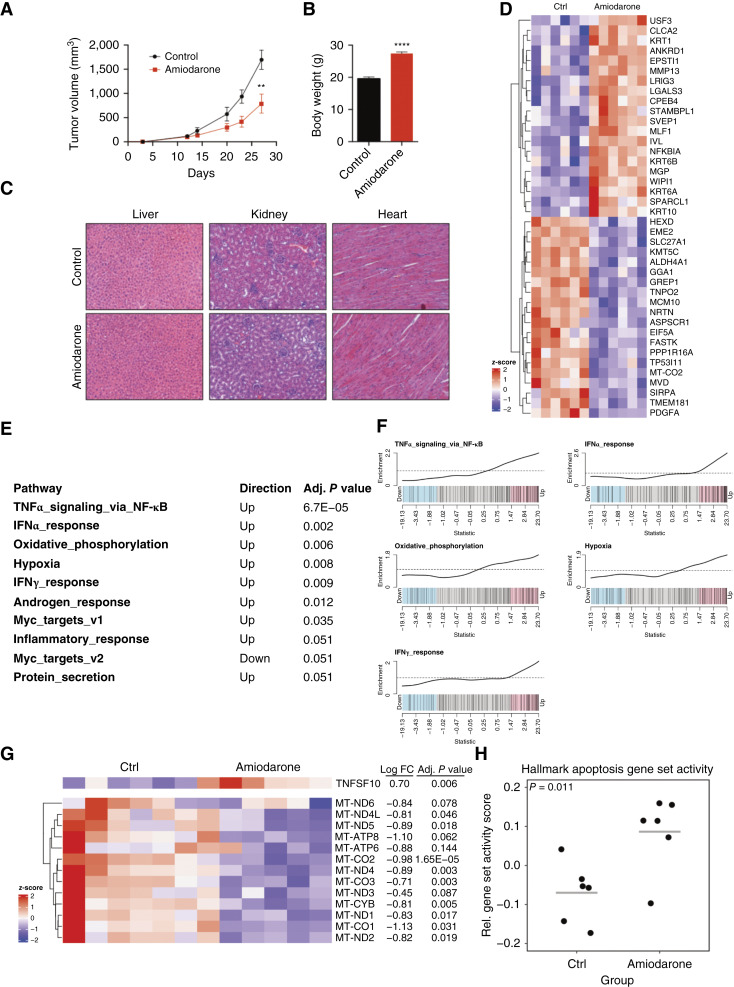
Amiodarone reduces bladder cancer tumor growth *in vivo*. Tumor growth in BFTC905-bearing NOD/SCID mice treated with 75 mg/kg of amiodarone. **A,** Tumor volume over time in control (*n* = 6) and amiodarone-treated (*n* = 6) tumor-bearing animals during a 3-week cycle treatment. Data represent mean ± SEM (Student *t* test; **, *P* < 0.01). **B,** Body weight of control and amiodarone-treated animals after 27 days of the experiment. Data represent mean ± SEM (Student *t* test; ****, *P* < 0.0001). **C,** Histologic analysis of liver, kidney, and heart sections derived from control and amiodarone-treated animals. Representative hematoxylin and eosin images are shown. **D,** Heatmap of the top 20 upregulated and top 20 downregulated genes identified by RNA-seq expression analysis of xenograft tumor samples. **E,** Top 10 significantly deregulated pathways identified by the RNA-seq analysis. **F,** Gene set enrichment analysis barcode plots for the top five significantly deregulated pathways. **G,** Heatmap showing the expression of the apoptosis-inducing ligand TNFSF10 and mitochondrially encoded genes of the electron transport chain. **H,** Dot plot representing the apoptosis gene set activity score derived from RNA-seq gene expression data. FC, fold change.

The role of amiodarone was investigated *in vivo* in immunosuppressed NOD/SCID mice bearing BFTC905 cells, which were one of the most sensitive cell lines to amiodarone in the *in vitro* studies. We established BFTC905 tumor-bearing mice as previously reported (33) and examined tumor growth and animal conditions for at least 3 weeks. The results indicated that tumor growth was significantly inhibited after treatment with 75 mg/kg amiodarone. Measurements on day 27 showed a 53.4% reduction in tumor volume in amiodarone-treated mice (790.89 ± 196.11 mm^3^) compared with control mice (1,696.49 ± 198.78 mm^3^; Fig. 5A). In addition, amiodarone treatment improved the health condition of the animals, which showed a higher body weight than untreated mice on day 27 (Fig. 5B).

Histologic analysis showed no differences in liver architecture between the treated and control mice, indicating no toxic effect of amiodarone at a concentration of 75 mg/kg (Fig. 5C). Similarly, no cardiotoxicity was observed following histochemical analysis of the hearts and kidneys when comparing treated and untreated mice (Fig. 5C). This indicates that amiodarone treatment resulted in an antitumor effect *in vivo* at a concentration of 75 mg/kg that was safe for the animals.

### *In vivo* effect of amiodarone on gene expression

RNA sequencing (RNA-seq) differential gene expression analysis of xenograft samples was conducted to investigate the molecular basis of amiodarone’s observed antitumor activity. This analysis revealed 798 significantly deregulated genes (347 upregulated and 451 downregulated), with the heatmap in Fig. 5D showing the top 20 upregulated and top 20 downregulated genes. Pathway analysis identified seven significantly altered pathways (adjusted *P* value < 0.05; Fig. 5E and F). Notably, we observed a significant enrichment of pathways associated with inflammatory responses, specifically TNFα signaling via NF-κB and IFNα/γ responses. Due to the lack of adaptive immunity in NOD/SCID mice, the observed inflammatory response likely results from a direct drug effect on the cancer cells. Further investigation revealed that TNFSF10 [TNF-related apoptosis-inducing ligand (TRAIL)], a key activator of tumor apoptosis, is significantly overexpressed (Fig. 5G). This suggests that amiodarone may lead to an apoptotic/inflammatory phenotype through autocrine activation of the TRAIL pathway.

Furthermore, pathway analysis highlighted the deregulation of energy-related pathways, including oxidative phosphorylation and hypoxia. A detailed analysis of the signatures and related genes revealed evidence of drug-induced mitochondrial dysfunction in the xenograft samples. Specifically, most of the mitochondrially encoded genes of the electron transport chain were significantly downregulated (Fig. 5G), potentially inducing hypoxia.

To validate the proapoptotic effect of amiodarone, we calculated an apoptosis gene set score (Gene Set Variation Analysis) for each individual sample. This analysis confirmed a significant upregulation of the apoptosis signature in treated samples (Fig. 5H). These results are in line with our *in vitro* analysis and confirm that amiodarone induces apoptosis in bladder cancer cells.

### Amiodarone inhibits mTOR and MAPK pathways in bladder cancer cell lines

The AKT/mTOR signaling pathway is crucial for cell survival and growth and has been implicated in bladder cancer tumorigenesis. As mentioned above, our RNA-seq data indicated possible induction of the hypoxic response after amiodarone treatment. It has been described that hypoxia may regulate mTOR induction in bladder cancer. In addition, desethylamiodarone (DEA), a metabolite of amiodarone, decreases AKT phosphorylation in cell lines from different types of cancers, including the bladder cancer cell line T24 (34). In T24 cells, DEA also decreased the phosphorylation of p44/42 MAPK (ERK1/2), which is a member of another central signaling pathway for cell proliferation.

**Figure 6:**
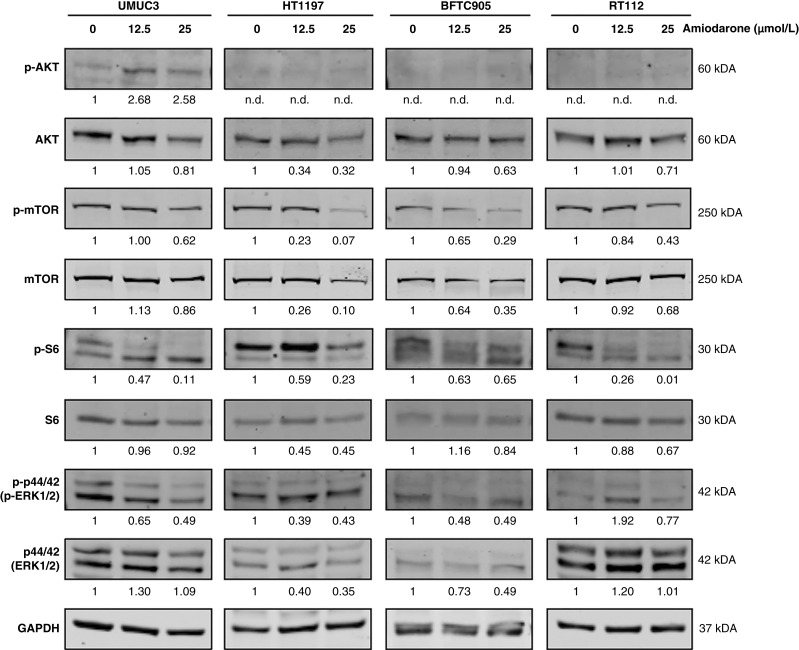
Amiodarone inhibits mTOR and MAPK pathways. Cells were treated for 24 hours with increasing concentrations of amiodarone (0, 12.5, and 25 μmol/L), and the expression of the proteins AKT, mTOR, S6, and p44/42 MAPK, both phosphorylated (p-) and total, was analyzed by Western blot. GAPDH was used as a loading control. Blots are representative of three independent experiments. Quantification of the protein expression (normalized by GAPDH) is indicated under each corresponding band. Values are presented as expression related to the control. n.d., not determined.

As described above, the effect of amiodarone on the phosphorylation state of key proteins in the AKT/mTOR pathway was analyzed. The basal levels of p-AKT were low in our four cell models, and no changes were detected after amiodarone treatment, except for UMUC3 cells, in which an increase in p-AKT was observed (Fig. 6). However, amiodarone downregulated p-mTOR and p-S6, two key downstream proteins of this pathway in the four cell lines. As S6 is one of the main targets of mTOR, our results suggest that amiodarone inhibits the mTOR pathway (Fig. 6). The effect of amiodarone on the phosphorylation of p44/42 MAPK was also analyzed. In line with previously reported data, amiodarone downregulated phospho-p44/42 in all cell lines except RT112, indicating an effect of the drug mainly in the invasive models (Fig. 6).

Our results showed that amiodarone inhibited the mTOR pathway in bladder cancer cell lines. In addition, amiodarone deactivated p44/42 MAPK, suggesting that the drug could also inhibit the MAPK pathway.

## Discussion

Platinum-based chemotherapy is an important therapeutic pillar in the treatment of MIBC in the perioperative setting or in metastatic disease (35). Following cisplatin treatment, cancer cells activate multiple DNA damage response pathways, such as nucleotide excision repair (8). This is one of the mechanisms involved in treatment resistance, which limits the patient’s response and highlights the necessity of developing new therapies for bladder cancer. Drug repurposing is a promising approach in cancer treatment, as it has a lower cost than the discovery of new drugs, and various studies have proposed the use of repurposed drugs in the context of bladder cancer. Recently, a high-throughput drug screen was performed in several bladder cancer cell lines, classified by cell subtypes, and identified clofarabine as one of the compounds with antineoplastic effects in cells from all of these subtypes (17). Clofarabine is an antimetabolite used in childhood acute lymphoblastic leukemia, and its effect against bladder cancer has been confirmed in cell lines, patient-derived cell cultures, and patient-derived cell xenograft models of urothelial and sarcomatoid carcinomas (17). However, the molecular mechanisms by which clofarabine inhibits bladder cancer growth remain unclear. Spironolactone, a drug approved for the treatment of hypertension, heart failure, acne, and hirsutism, has been identified as a potential repurposed drug for bladder cancer (18). It was found that spironolactone inhibits the expression of excision repair cross-complementation 3, reduces bladder cancer growth, and enhances platinum cytotoxicity *in vitro* and *in vivo* (18). Similarly, disulfiram, a drug used in patients with alcoholic disorders, was found to enhance sensitivity to cisplatin in bladder cancer xenograft models (15). The potential of cationic amphiphilic drugs, such as penfluridol, in repurposing has been identified by van der Horst and colleagues (20). Treatment with these drugs leads to lysosome-dependent cell death and growth inhibition in human bladder cancer models *in vivo*. Some cell lines used in our study were also sensitive to spironolactone, disulfiram, and penfluridol. In summary, drug repurposing has emerged as a promising approach for bladder cancer treatment, with the potential application of drugs in combination with existing therapies.

In the present study, we expanded our previously established pipeline, based on the integration of multiomics data from hundreds of bladder cancer (including both NMIBC and MIBC) specimens, to obtain a list of 27 compounds with potential antitumor activity against aggressive bladder cancer. We focused on the integrative analysis of changes between MIBC and NMIBC. This is rooted in the current clinical framework and our previous transcriptomics research (29) that has demonstrated that there are continuous and progressive molecular changes across different stages of bladder cancer. This monotonicity in molecular alterations led us to hypothesize that the transition from NMIBC to MIBC is not a sudden or discrete event but rather a continuum of molecular changes. Thus, therapies effective against the advanced stage of MIBC may also demonstrate efficacy in earlier stages, such as NMIBC. However, the significance of molecular heterogeneity cannot be underestimated, particularly with the recognition of various molecular subtypes in recent years. To better address the molecular heterogeneity and potential differences in treatment response, we employed a multiomics approach, enabling us to capture a broader molecular landscape across disease phenotypes. The application of this multiomics approach seems to enhance the identification of predicted drug candidates. A direct comparison between our previous studies and the current one is not feasible due to differences in the methodologies used to define the molecular signatures. Although the previous study focused on proteomic differences among various NMIBC subtypes, the current study investigates proteotranscriptomic differences between MIBC and NMIBC. Nonetheless, the success of the current pipeline is evident in its identification of a substantial number of FDA-approved drugs that, when repurposed for bladder cancer, demonstrate efficacy both *in vitro* and *in vivo*. However, it is important to note that the CMap platform is not designed to account for specific mutations. Therefore, potential therapeutic resistance related to mutational changes has not been investigated in this analysis. Other studies have reported the clinical potential of repurposed antibiotics in bladder cancer. For example, the use of β-lactam antibiotics in patients with early-stage bladder cancer did not alter the recurrence rate (36). Using *in vitro* viability assays, we demonstrated that our *in silico* drug repurposing approach was successful: Four of the predicted candidates—amiodarone, amodiaquine, fluvoxamine, and isradipine (∼15% of the total)—decreased cell viability in a group of different bladder cancer cell lines. Interestingly, the most potent candidate against bladder cancer is amiodarone, a widely used class III antiarrhythmic drug. First, viability assays using a colorimetric method showed a dose-dependent effect of amiodarone on our four cell models, with IC_50_ values in the range of 5.38 to 22.85 μmol/L. Proliferation assays using an Incucyte system with the same cell lines confirmed previous results, revealing the IC_50_ values for amiodarone in the range of 5.02 to 16.22 μmol/L. These values were obtained 96 hours after treatment with the drug; however, the effect of increasing concentrations of amiodarone on cell proliferation was evident in the early stages of the experiment. The IC_50_ values for amiodarone at 48 and 72 hours in every cell line were similar to those obtained at 96 hours. Moreover, the treatment of the benign bladder cell HBLAK with amiodarone concentrations up to 20 μmol/L did not result in an antiproliferative effect, indicating that the effect of the drug is bladder cancer cell–specific. In line with the proliferation results, low concentrations of amiodarone reduced the colony formation capacity in a dose-dependent manner in the four cell lines. Interestingly, the invasive cell lines HT1197 and BFTC905 were the most sensitive to amiodarone in both proliferation and colony formation assays. The most notable effect was on the clonogenic capacity, which was strongly reduced (>85%) with 3 μmol/L amiodarone and completely abolished with 5 μmol/L of the drug. HT1197 and BFTC905 have been classified as basal subtype cell lines and represent tumor stages pT2 and pT4, respectively (31, 37). Basal bladder cancer cell lines could be treated with MEK inhibitors, as confirmed by our signal transduction analysis (38). UMUC3 has been classified as a neuronal basal cell line representing stages pT2 to pT4 (31). In contrast, RT112 is a luminal cell line that represents stage pTa cancer (31). Based on this, amiodarone had a stronger effect on the most aggressive cell models, validating our *in silico* strategy for predicting this drug as a candidate for MIBC. Our results are in line with previous studies showing an anticancer effect of amiodarone *in vitro* in other types of cancer, such as cervical cancer, glioma, and leukemia (39–41). This was demonstrated by viability assays, and two of these studies also showed that amiodarone inhibited colony formation in the HeLa (cervical cancer) and U-87 MG (glioblastoma) cell lines (40, 41). In addition, a separate study reported that DEA, a metabolite of amiodarone, reduces viability and colony formation in cell lines from different types of cancers: T24 (bladder), HeLa (cervical), B16F10 (mouse melanoma), and MCF-7 and 4T1 (human and mouse breast, respectively; refs. 34, 42).

Amiodarone induced cell death in our four bladder cancer cell models through caspase 3 and 7 activation. This was confirmed by Western blot assays showing increased levels of the apoptosis marker cPARP after treatment with amiodarone. Interestingly, we did not detect cPARP expression in BFTC905 cells treated with amiodarone although this cell line had the highest caspase 3/7 activation. Differences in the detection time points and types of methodologies could explain this discrepancy, highlighting the importance of using more than one method for confirming cell death. It has been reported that amiodarone sensitizes glioma cells to TRAIL-induced cell death via caspase-dependent apoptosis (43) and activates intrinsic apoptosis in HeLa cells (40). Similarly, amiodarone induced apoptosis in myeloid leukemia cells, and the combination with the BH3 mimetic ABT-263 enhanced apoptosis (39). Moreover, amiodarone increased the susceptibility of a glioblastoma cell line to anoikis, a form of programmed cell death produced when cells detach from the extracellular matrix (41). In addition, studies using DEA have shown that this metabolite induces apoptosis in cell lines from different types of cancers, including the bladder cancer cell line T24. Regarding other repurposing studies on bladder cancer and similar to our results on amiodarone, the induction of apoptosis by spironolactone (18), disulfiram (15), and penfluridol (20) were reported in bladder cancer cell lines.

In line with our *in vitro* results, amiodarone reduced tumor growth in a xenograft bladder cancer mouse model, confirming the initially predicted anticancer activity of this drug. Moreover, we showed, for the first time, the antitumor effect of amiodarone against bladder cancer *in vivo*. This is in line with the few studies that have analyzed the effects of amiodarone on other types of cancers in mouse models. Steinberg and colleagues (41) showed that amiodarone inhibits tumor growth and the vasculature of glioblastoma xenografts. The reduction in tumor volume in treated mice was observed with a notably lower concentration of amiodarone (0.1 mg/kg/day) compared with the one we used. However, in the aforementioned study, the route of administration was i.p. injection, whereas oral administration was used in our experiments. It has been described that i.p. administration of drugs results in faster and more complete absorption compared with oral administration (44). Using a mouse metastasis model, Bognar and colleagues (34) showed that DEA reduced lung metastasis of B16-F10 melanoma cells when 25 mg/kg of this metabolite was administered by intraperitoneally every 3 days. Employing a similar experimental model, Lee and colleagues (45) reported that amiodarone inhibited lung metastasis of murine breast cancer cells 4T-1 when mice were treated with 180 mg/kg/day of amiodarone orally. Here, the route and frequency of amiodarone administration were the same as those in our study; however, we observed an antitumor effect with a lower concentration of the drug (75 mg/kg/day). In a clinical context, amiodarone is administered to patients with ventricular arrhythmias at doses of more than 1,000 mg per day intravenously, followed by 400 to 1,600 mg per day orally, depending on the duration of intravenous treatment. Based on Reagan-Shaw’s conversion formula (46), 75 mg/kg of amiodarone used in mice is equivalent to 365 mg per day in an adult human. This is lower than the doses usually administered to cardiac patients, suggesting that amiodarone could be used for bladder cancer treatment at a concentration that is generally safe.

Based on RNA-seq data, we have highlighted seven signaling pathways that may play a role in the modulation of the amiodarone effect. In this context, it is known that IFN could antagonize the oncogenic effect of the transcription factor FOXA1 in bladder cancer (47). Similarly, it was recently reported that IFNγ induces apoptosis in urothelial cancer, thus modulating the effect of the anti–PD-1 antibody pembrolizumab (48). Synergistic induction of apoptosis by amiodarone and TRAIL was observed in various glioma cells (43). The results of RNA-seq reported in the present study are in agreement with those of Krajcova and colleagues (49). They reported the induction of mitochondrial stress by amiodarone in cardiomyocytes and liver cells. The increase in the OxPhos signature may represent a compensatory mechanism to cope with the lack of cellular energy.

The AKT/mTOR pathway has been implicated in bladder cancer tumorigenesis, and previous studies in other types of cancers have shown that amiodarone downregulates AKT activity. It has been reported that amiodarone reduces p-AKT in the murine cancer cell lines B16OVA (melanoma) and JC and 4T-1 (both murine breast cancer) as well as in the human breast cancer cell line MDA-MB231. In addition, one of the target proteins of the pathway, GSK-3β, was also reduced, which has been proposed as a mechanism for repressing epithelial-to-mesenchymal transition (45). Reduction in p-AKT after treatment with amiodarone was also reported in two myeloid leukemia cell lines, with a stronger reduction observed when amiodarone was combined with ABT-263 (39). DEA showed similar effects in reducing p-AKT in cell lines from different types of cancers, including the bladder cancer cell line T24, in which GSK-3β was also reduced (34). Interestingly, in the present study, we observed low basal levels of p-AKT in the four cell models. In contrast to the literature, increased p-AKT levels were detected with amiodarone treatment in UMUC3 cells, whereas no changes were observed in the other cell lines. However, amiodarone reduced the phosphorylation of the two main downstream proteins of AKT, mTOR, and S6 in our four cell models. These results indicate that amiodarone inhibits *in vitro* the mTOR pathway, which acts as a sensor of mitochondrial function without affecting AKT in most of the cell lines. Thus, we hypothesize that inhibition of the mTOR pathway in bladder cancer cells is a secondary phenomenon following mitochondrial stress and TRAIL regulation. The upregulation of p-AKT induced by amiodarone in UMUC3 is a phenomenon that has been previously described for mTOR inhibitors, such as rapamycin (50). This inhibitor can activate AKT through a negative feedback loop in the pathway (50), which could explain our results in UMUC3. Indeed, the effect of rapamycin on this cell line has been reported, showing that rapamycin decreases p-S6 and increases p-AKT levels (51), suggesting that amiodarone can act similarly to rapamycin in UMUC3 cells. In addition to the described increase in p-AKT in some cancer cell lines, rapamycin produced no change or decreased p-AKT in other cell lines (52). This differential effect between cell types seems to be similar to that observed for amiodarone in our cell models. In conclusion, in addition to the effects of amiodarone on AKT, the drug consistently reduced p-mTOR and p-S6 in the four bladder cancer cell lines, suggesting that the anticancer effect of amiodarone in bladder cancer is mediated by the inhibition of mTOR. Considering the complexity of the mTOR signaling pathway, further studies focusing on the different members of the mTOR complex and other proteins involved in the pathway are needed to identify specific targets of amiodarone in bladder cancer. With regard to differences in mTOR regulation by amiodarone between *in vitro* and *in vivo* experiments, one must keep in mind that the duration of the *in vivo* treatment with amiodarone was much longer compared with cell culture experiments. For example, mTOR activity in acute myeloid leukemia may decrease during tumor progression (53). Thus, the fact that the effect of amiodarone on mTOR has not been observed in urothelium tumor material is not necessarily surprising. Clinical studies may investigate the question of whether amiodarone could be used in bladder cancer treatment without limiting side effects. We showed that the phosphorylation of p44/42 (ERK1/2) was also decreased by amiodarone, which is in line with previous studies in other types of cancer. Lee and colleagues (45) reported similar effects in melanoma and breast cancer cell lines. In addition, DEA decreased p-ERK1/2 levels in B16F10 mouse melanoma cells and T24 cells.

In summary, we demonstrated that our *in silico* repurposing pipeline was successful in predicting candidates with antitumor activity for bladder cancer. Our most promising candidate, amiodarone, reduced bladder cancer growth *in vitro* and *in vivo*, an effect that might be mediated by the inhibition of mTOR, with possible implications also for the ERK1/2 pathway. Thus, we propose amiodarone as a potential repurposed drug for bladder cancer treatment, especially for MIBC. Furthermore, the use of repurposed drugs for bladder cancer may be a useful approach for specific molecular subgroups of patients. Recently, the IL-6/signal transducer and activator of transcription factor 3 axis has been identified as a target in a subgroup of bladder cancer (54).

In future preclinical studies, combinatorial therapies using amiodarone and targeted therapies or immunotherapies could be tested in models similar to those used in the present study or in novel patient-derived xenograft models (54).

## Supplementary Material

Supplementary Figure 1 Supplementary Figure 1. Fluvoxamine, amiodarone, isradipine, and amodiaquine reduce viability in BC cell lines. A, UMUC3, HT1197, BFTC905, and RT112 cells were treated for 96 h with increasing concentrations (0–100 μM) of fluvoxamine (A), amiodarone (B), isradipine (C), and amodiaquine (D). Viability was evaluated using the CellTiter Cell Proliferation Assay. Concentration-response curves and IC50 values are shown. Data represent mean ± SEM from 3 independent experiments. h, hour; n.d., not determined.

Supplementary Figure 2 Supplementary Figure 2. The effect of amiodarone is bladder cancer specific. Real-time proliferation assays in the benign bladder cells HBLAK using the IncucyteS3 System. The cells were treated with increasing concentrations of amiodarone (0-50 μM) and the confluence was measured every 4 h during 96 h. A, Cell confluence over time. B, Cell confluence after 96 h treatment. Data represent mean ± SEM from 3 independent experiments (one-way ANOVA with Dunnett’s multiple comparison test; ****P < 0.0001). C, Concentration-response curve for amiodarone after 96 h treatment. h, hour; n.d., not determined.

Supplementary Figure Legends File contains 2 Supplementary Figure legends

Supplementary Table 1 Differentially expressed genes (DEGs) between muscle invasive bladder cancer (MIBC) and non-muscle invasive bladder cancer (NBIMC).

Supplementary Table 2 List of differentially abundant proteins between muscle invasive bladder cancer (MIBC) and non-muscle invasive bladder cancer (NMIBC)

Supplementary File 1 The analysis based on the previously generated proteomics data

Supplementary File 2 Gene expression results
