# Supplementary figures and images for: Repurposing Amiodarone for Bladder Cancer Treatment
Source: Cancer Res Commun. 2025 Jun 4;5(6):906–20. doi: 10.1158/2767-9764.CRC-24-0433 (PMC12134865; doi:10.1158/2767-9764.CRC-24-0433)

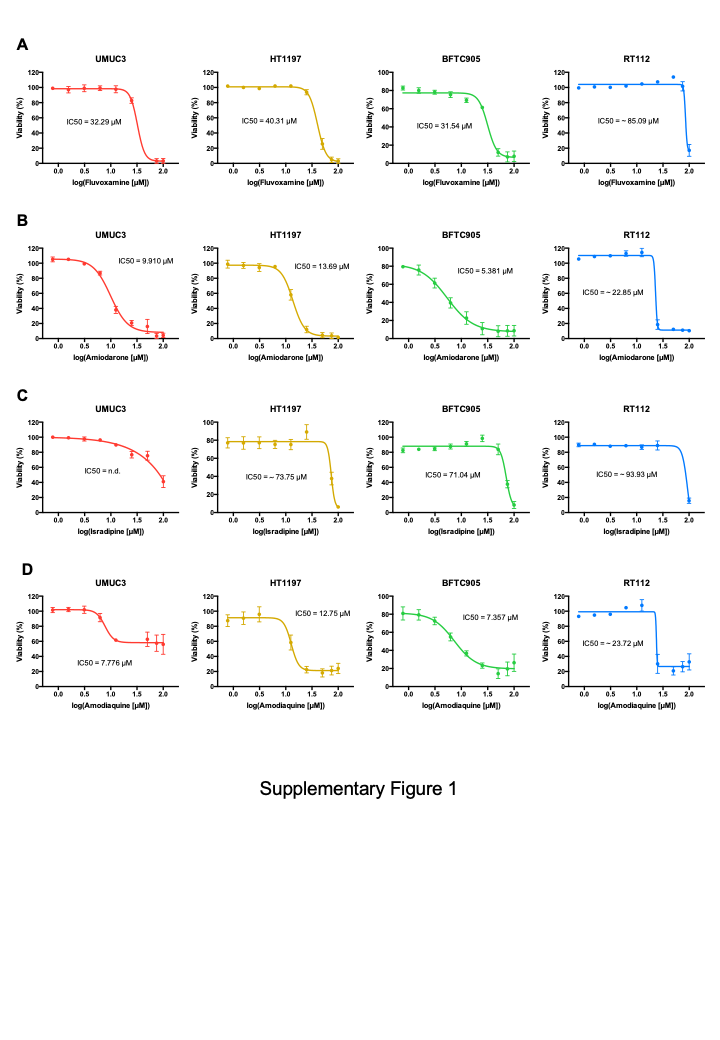

Supplement: Supplementary Figure 1 — Fluvoxamine, amiodarone, isradipine, and amodiaquine reduce viability in BC cell lines. A, UMUC3, HT1197, BFTC905, and RT112 cells were treated for 96 h with increasing concentrations (0–100 μM) of fluvoxamine (A), amiodarone (B), isradipine (C), and amodiaquine (D). Viability was evaluated using the CellTiter Cell Proliferation Assay. Concentration-response curves and IC50 values are shown. Data represent mean ± SEM from 3 independent experiments. h, hour; n.d., not determined. [file crc-24-0433_supplementary_figure_1_supp1.png]

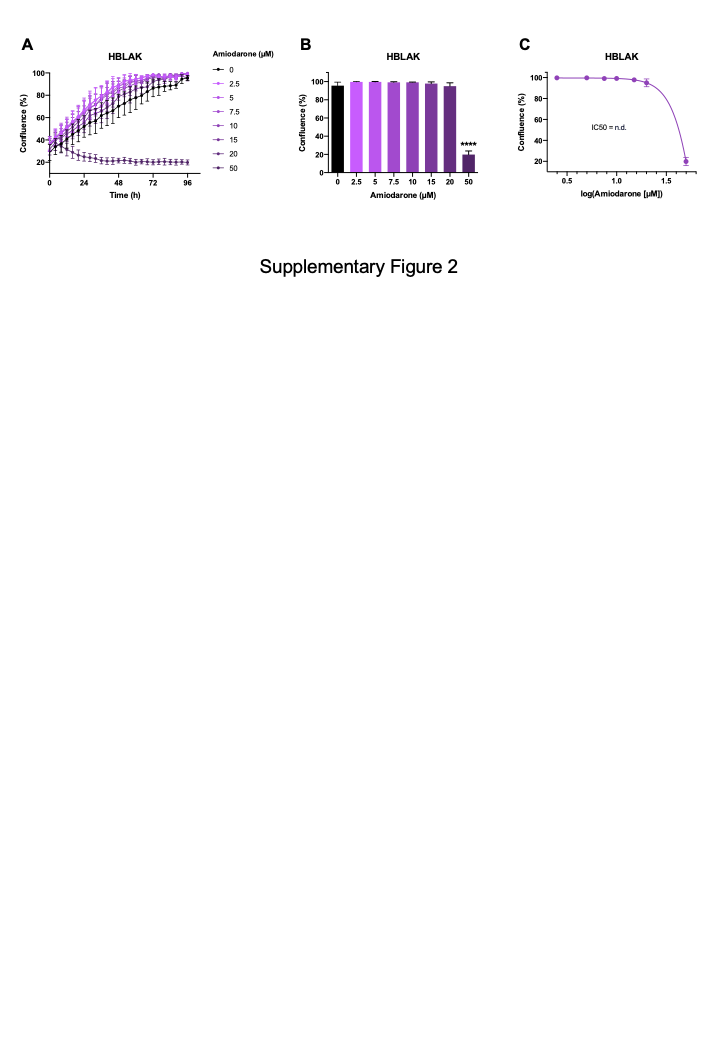

Supplement: Supplementary Figure 2 — The effect of amiodarone is bladder cancer specific. Real-time proliferation assays in the benign bladder cells HBLAK using the IncucyteS3 System. The cells were treated with increasing concentrations of amiodarone (0-50 μM) and the confluence was measured every 4 h during 96 h. A, Cell confluence over time. B, Cell confluence after 96 h treatment. Data represent mean ± SEM from 3 independent experiments (one-way ANOVA with Dunnett’s multiple comparison test; ****P < 0.0001). C, Concentration-response curve for amiodarone after 96 h treatment. h, hour; n.d., not determined. [file crc-24-0433_supplementary_figure_2_supp2.png]
