# Supplementary material for: Repurposing Amiodarone for Bladder Cancer Treatment
Source: Cancer Res Commun. 2025 Jun 4;5(6):906–20. doi: 10.1158/2767-9764.CRC-24-0433 (PMC12134865; doi:10.1158/2767-9764.CRC-24-0433)
Supplement: Supplementary Figure Legends — File contains 2 Supplementary Figure legends [file crc-24-0433_supplementary_figure_legends_suppsfl.docx]

**SUPPLEMENTARY FIGURE LEGENDS**

**Supplementary Figure 1. Fluvoxamine, amiodarone, isradipine, and amodiaquine reduce viability in BC cell lines.** **A,** UMUC3, HT1197, BFTC905, and RT112 cells were treated for 96 h with increasing concentrations (0-100 μM) of fluvoxamine (A), amiodarone (B), isradipine (C), and amodiaquine (D). Viability was evaluated using the CellTiter Cell Proliferation Assay. Concentration-response curves and IC50 values are shown. Data represent mean ± SEM from 3 independent experiments. *h*, hour; *n.d.*, not determined.

**Supplementary Figure 2. The effect of amiodarone is bladder cancer specific.** Real-time proliferation assays in the benign bladder cells HBLAK using the IncucyteS3 System. The cells were treated with increasing concentrations of amiodarone (0-50 μM) and the confluence was measured every 4 h during 96 h. **A,** Cell confluence over time. **B,** Cell confluence after 96 h treatment. Data represent mean ± SEM from 3 independent experiments (one-way ANOVA with Dunnett’s multiple comparison test; ****P < 0.0001). **C,** Concentration-response curve for amiodarone after 96 h treatment. *h*, hour; *n.d.*, not determined.
