# Supplementary material for: Repurposing Amiodarone for Bladder Cancer Treatment
Source: Cancer Res Commun. 2025 Jun 4;5(6):906–20. doi: 10.1158/2767-9764.CRC-24-0433 (PMC12134865; doi:10.1158/2767-9764.CRC-24-0433)
Supplement: Supplementary Table 1 — Differentially expressed genes (DEGs) between muscle invasive bladder cancer (MIBC) and non-muscle invasive bladder cancer (NBIMC). [file crc-24-0433_supplementary_table_1_suppst1.pdf]

**Supplementary Table 1.** Differentially expressed genes (DEGs) between muscle invasive bladder cancer (MIBC) and non-muscle invasive bladder cancer (NBIMC).			
**Symbol**	**BH p-value**	**AUC**	**Change (MIBC vs NMIBC)**
ABAT	1.01E-10	**0.63**	-0.35
ABCC3	2.01E-13	**0.64**	-0.42
ACAA1	4.94E-09	**0.61**	-0.16
ACBD4	2.27E-08	**0.61**	-0.26
ACOT11	2.75E-09	**0.62**	-0.21
ACOXL	1.45E-11	**0.63**	-0.55
ACP6	2.48E-11	**0.63**	-0.28
ACSL5	4.26E-12	**0.63**	-0.39
ACTA2	3.78E-08	**0.61**	0.32
ACTG2	9.73E-11	**0.63**	0.50
ACTN1	2.32E-15	**0.65**	0.37
ADA	7.84E-08	**0.61**	0.22
ADAM19	1.70E-16	**0.66**	0.37
ADAMTS2	1.48E-09	**0.62**	0.33
ADCY6	1.82E-10	**0.62**	-0.18
AEBP1	4.21E-16	**0.66**	0.47
AIF1	3.17E-10	**0.62**	0.25
ALDH1L2	1.48E-13	**0.64**	0.33
ALDH4A1	2.39E-09	**0.62**	-0.23
ALOX5	8.06E-12	**0.63**	-0.38
ALOX5AP	8.99E-09	**0.61**	0.29
ANXA1	1.20E-10	**0.63**	0.49
ANXA10	5.10E-14	**0.65**	-0.85
ANXA2	1.40E-08	**0.61**	0.22
ANXA5	1.89E-13	**0.64**	0.40
AOAH	2.34E-07	**0.60**	0.24
AP1G2	6.79E-11	**0.63**	-0.23
AP2S1	1.59E-08	**0.61**	0.11
APCDD1L	6.69E-09	**0.61**	0.33
APOE	1.27E-07	**0.60**	0.26
APOL1	8.69E-11	**0.63**	-0.37
ARFGEF1	3.19E-07	**0.60**	-0.14
ARHGEF10L	1.42E-10	**0.62**	-0.35
ARHGEF12	1.61E-07	**0.60**	-0.14
ARSD	7.56E-11	**0.63**	-0.25
ASCC2	1.21E-10	**0.63**	-0.23
ATF7IP2	3.69E-08	**0.61**	-0.29
ATOH8	4.79E-11	**0.63**	-0.47
ATP8B1	1.05E-12	**0.64**	-0.37
AXL	6.70E-08	**0.61**	0.27
B3GALNT1	6.68E-11	**0.63**	-0.29
BAZ2A	2.50E-07	**0.60**	-0.17
BCAS1	2.47E-16	**0.66**	-0.69

BCAT1	5.89E-14	**0.65**	0.43
BCKDHB	2.30E-09	**0.62**	-0.22
BGN	7.52E-08	**0.61**	0.29
BTBD16	1.17E-14	**0.65**	-0.72
BTBD8	1.39E-07	**0.60**	-0.23
BVES	1.24E-08	**0.61**	0.25
C10orf99	1.21E-10	**0.63**	-0.65
C15orf48	2.40E-08	**0.61**	0.39
C1orf162	7.86E-08	**0.61**	0.20
C1QA	2.94E-09	**0.62**	0.38
C1QB	1.24E-10	**0.63**	0.38
C1QC	3.43E-11	**0.63**	0.38
C1R	4.95E-13	**0.64**	0.36
C5AR1	5.48E-11	**0.63**	0.33
CALD1	3.18E-15	**0.65**	0.50
CALU	3.18E-15	**0.65**	0.27
CAPN5	8.06E-12	**0.63**	-0.33
CAPS	5.19E-10	**0.62**	-0.33
CARD11	4.04E-13	**0.64**	-0.32
CAV1	1.07E-08	**0.61**	0.35
CBR4	6.49E-10	**0.62**	-0.22
CCDC80	7.10E-08	**0.61**	0.30
CCL11	2.96E-08	**0.61**	0.33
CCL2	7.16E-13	**0.64**	0.38
CCL8	2.04E-10	**0.62**	0.37
CD109	7.55E-09	**0.61**	0.39
CD14	1.68E-12	**0.64**	0.36
CD163	2.94E-11	**0.63**	0.35
CD248	1.66E-07	**0.60**	0.22
CD86	7.89E-11	**0.63**	0.26
CD96	3.37E-08	**0.61**	-0.22
CDC25B	1.32E-14	**0.65**	0.32
CDCA5	1.14E-08	**0.61**	0.25
CDH11	4.34E-11	**0.63**	0.39
CDH23	6.58E-09	**0.61**	-0.34
CDK6	1.55E-07	**0.60**	0.33
CEBPB	2.38E-07	**0.60**	0.19
CENPA	5.49E-08	**0.61**	0.25
CEP290	3.03E-07	**0.60**	-0.12
CEP55	1.08E-07	**0.60**	0.27
CIRBP	1.90E-08	**0.61**	-0.17
CLCA4	1.02E-07	**0.60**	-0.60
CLCN3	2.07E-09	**0.62**	-0.18
CLIC4	6.60E-18	**0.67**	0.40
CLIP3	1.49E-07	**0.60**	0.16
CNN1	1.24E-09	**0.62**	0.50
CNN3	5.99E-11	**0.63**	0.29
CNTN1	7.08E-08	**0.61**	0.34
CNTN3	6.68E-11	**0.63**	-0.52
COL10A1	2.35E-10	**0.62**	0.48

COL11A1	3.53E-09	**0.62**	0.46
COL12A1	7.85E-14	**0.65**	0.40
COL15A1	7.41E-10	**0.62**	0.30
COL16A1	2.20E-10	**0.62**	0.30
COL1A1	1.79E-17	**0.67**	0.48
COL1A2	4.21E-16	**0.66**	0.48
COL3A1	3.96E-17	**0.66**	0.50
COL5A1	3.18E-15	**0.65**	0.40
COL5A2	8.58E-17	**0.66**	0.45
COL6A1	6.14E-12	**0.63**	0.38
COL6A2	1.61E-12	**0.64**	0.41
COL6A3	4.21E-16	**0.66**	0.50
COL8A1	3.96E-08	**0.61**	0.28
COLEC12	2.39E-07	**0.60**	0.27
COMP	6.07E-09	**0.61**	0.48
COMT	2.53E-09	**0.62**	-0.20
COPZ2	3.12E-10	**0.62**	0.22
CORO1C	3.21E-13	**0.64**	0.30
COX4I1	1.12E-07	**0.60**	-0.10
CPVL	1.93E-10	**0.62**	0.27
CPXM2	6.39E-08	**0.61**	0.27
CRISPLD2	4.41E-09	**0.61**	0.26
CROT	4.70E-08	**0.61**	-0.25
CRTAC1	1.76E-13	**0.64**	-0.89
CRYAB	4.82E-08	**0.61**	0.34
CSF1R	9.57E-08	**0.60**	0.22
CTHRC1	2.11E-18	**0.67**	0.51
CTPS2	6.37E-09	**0.61**	-0.16
CTSE	5.57E-11	**0.63**	-0.62
CTSH	6.86E-09	**0.61**	-0.31
CTSK	1.60E-14	**0.65**	0.39
CXCL10	3.05E-08	**0.61**	0.49
CXCL2	1.70E-07	**0.60**	0.32
CXXC1	1.10E-08	**0.61**	-0.15
CYBRD1	1.18E-08	**0.61**	0.30
CYP1B1	1.59E-10	**0.62**	0.38
CYP2J2	9.39E-10	**0.62**	-0.42
CYP3A5	2.22E-13	**0.64**	-0.51
CYP4B1	2.69E-10	**0.62**	-0.71
CYP4F12	4.66E-11	**0.63**	-0.50
DACT1	4.12E-09	**0.61**	0.31
DCN	1.49E-11	**0.63**	0.44
DECR1	1.69E-11	**0.63**	-0.19
DEGS1	4.98E-18	**0.67**	0.33
DENND2D	1.73E-14	**0.65**	-0.32
DEPDC7	5.16E-08	**0.61**	0.28
DGKA	9.94E-08	**0.60**	-0.21
DIO2	9.83E-11	**0.63**	0.36
DISP1	1.50E-07	**0.60**	-0.13
DNAJA4	2.05E-13	**0.64**	-0.28

DNM1	1.11E-07	**0.60**	0.30
DNM2	2.20E-10	**0.62**	-0.21
DOCK11	8.25E-10	**0.62**	0.26
DOCK2	2.53E-07	**0.60**	0.21
DPYSL2	6.40E-08	**0.61**	0.23
DPYSL3	1.63E-12	**0.64**	0.39
DSC2	1.12E-07	**0.60**	0.40
DSE	2.57E-12	**0.64**	0.35
DUSP14	9.39E-10	**0.62**	0.20
EDARADD	7.52E-08	**0.61**	-0.21
EDNRA	3.23E-08	**0.61**	0.22
EFEMP1	8.06E-12	**0.63**	0.50
EHD1	9.21E-08	**0.60**	0.13
EIF5A2	1.09E-07	**0.60**	0.19
ELOVL4	2.40E-07	**0.60**	0.17
ELOVL5	1.35E-07	**0.60**	0.22
EMILIN1	9.74E-09	**0.61**	0.28
EMP1	2.40E-07	**0.60**	0.27
EMP3	3.88E-13	**0.64**	0.34
ENTPD3	9.71E-09	**0.61**	-0.35
ERBB3	6.65E-10	**0.62**	-0.32
EXOC7	3.72E-07	**0.60**	-0.13
FAAH	1.06E-07	**0.60**	-0.19
FABP6	8.77E-13	**0.64**	-0.51
FAM20C	1.44E-12	**0.64**	0.30
FAM3B	2.90E-09	**0.62**	-0.54
FAM3D	2.30E-09	**0.62**	-0.48
FAM83D	9.02E-10	**0.62**	0.33
FAP	2.41E-18	**0.67**	0.53
FBLN2	1.89E-07	**0.60**	0.28
FBN1	4.33E-09	**0.61**	0.35
FBP1	5.67E-12	**0.63**	-0.50
FBXO32	1.59E-09	**0.62**	0.28
FBXW4	1.35E-07	**0.60**	-0.13
FCER1G	5.75E-12	**0.63**	0.36
FCGR2A	8.99E-09	**0.61**	0.21
FEN1	2.23E-08	**0.61**	0.18
FER1L4	2.06E-12	**0.64**	-0.45
FGFR3	2.18E-09	**0.62**	-0.44
FHIT	1.18E-10	**0.63**	-0.32
FILIP1L	4.27E-12	**0.63**	0.29
FKBP5	1.66E-07	**0.60**	0.25
FLNA	4.31E-09	**0.61**	0.35
FLNC	3.13E-09	**0.62**	0.38
FLOT2	1.71E-07	**0.60**	-0.13
FMNL2	1.20E-07	**0.60**	0.31
FN1	3.55E-13	**0.64**	0.48
FNBP1L	1.71E-07	**0.60**	-0.23
FNDC1	9.31E-12	**0.63**	0.46
FOXA1	4.38E-08	**0.61**	-0.41

FOXQ1	1.95E-08	**0.61**	-0.37
FPR1	3.72E-10	**0.62**	0.35
FSTL1	2.09E-11	**0.63**	0.28
FXYD6	2.28E-07	**0.60**	0.29
GAS1	1.66E-12	**0.64**	0.45
GAS6	5.67E-08	**0.61**	0.25
GATA2	1.55E-10	**0.62**	-0.37
GEM	1.84E-09	**0.62**	0.32
GEMIN8	4.92E-09	**0.61**	-0.17
GFOD1	1.00E-08	**0.61**	0.19
GFPT2	2.31E-17	**0.66**	0.58
GGT6	1.93E-10	**0.62**	-0.41
GLI2	7.49E-08	**0.61**	0.25
GLIPR1	2.03E-07	**0.60**	0.29
GLT8D2	2.54E-09	**0.62**	0.23
GNB4	1.65E-07	**0.60**	0.26
GNPDA1	2.67E-07	**0.60**	-0.14
GOLT1A	4.38E-08	**0.61**	-0.36
GPC6	2.50E-08	**0.61**	0.16
GPNMB	3.93E-09	**0.62**	0.43
GPR68	1.07E-07	**0.60**	0.28
GPRC5C	3.30E-12	**0.64**	-0.31
GPX2	7.71E-08	**0.61**	-0.54
GREM1	1.24E-16	**0.66**	0.60
GYPC	6.74E-08	**0.61**	0.19
HAPLN3	2.25E-09	**0.62**	0.29
HARS2	1.35E-08	**0.61**	-0.12
HAS2	3.46E-07	**0.60**	0.30
HAS3	3.82E-08	**0.61**	-0.34
HAVCR2	2.73E-08	**0.61**	0.20
HCK	6.42E-09	**0.61**	0.27
HERC5	6.04E-08	**0.61**	0.24
HHAT	7.23E-09	**0.61**	-0.27
HMGCS2	3.55E-13	**0.64**	-0.87
HOXB5	2.43E-09	**0.62**	-0.32
HOXB6	1.66E-07	**0.60**	-0.34
HS3ST1	6.71E-08	**0.61**	-0.26
HS3ST3A1	1.40E-09	**0.62**	0.31
HSD17B2	7.39E-14	**0.65**	-0.63
HSD17B6	2.82E-10	**0.62**	0.31
ID1	1.62E-13	**0.64**	-0.42
ID2	4.01E-09	**0.62**	-0.27
ID3	4.27E-10	**0.62**	-0.29
ID4	3.50E-09	**0.62**	-0.35
IFITM2	6.50E-09	**0.61**	0.28
IFT140	2.47E-07	**0.60**	-0.23
IGBP1	3.19E-07	**0.60**	-0.12
IKZF2	4.38E-08	**0.61**	-0.32
IL32	1.22E-10	**0.63**	0.31
ILDR1	1.53E-07	**0.60**	-0.26

IMPA2	9.53E-11	**0.63**	0.28
INA	5.81E-08	**0.61**	-0.45
ING4	2.33E-07	**0.60**	-0.11
IRAG2	2.40E-07	**0.60**	-0.24
ISLR	5.31E-16	**0.66**	0.53
ITFG2	2.59E-07	**0.60**	-0.19
ITGA11	3.42E-08	**0.61**	0.31
ITGA5	9.50E-12	**0.63**	0.33
KATNAL1	5.17E-11	**0.63**	0.35
KCNJ8	4.01E-09	**0.61**	0.21
KCNQ1	5.67E-08	**0.61**	-0.16
KDELR3	2.92E-08	**0.61**	0.25
KLHL5	3.22E-10	**0.62**	0.27
LAMA4	9.35E-08	**0.60**	0.22
LATS2	1.15E-07	**0.60**	0.19
LCOR	3.63E-12	**0.64**	-0.20
LGALS1	9.50E-15	**0.65**	0.39
LIX1L	1.99E-07	**0.60**	0.16
LMCD1	1.61E-11	**0.63**	0.26
LNX1	2.29E-11	**0.63**	-0.42
LOX	9.02E-16	**0.66**	0.50
LRBA	1.78E-07	**0.60**	-0.16
LRIG1	4.07E-11	**0.63**	0.35
LUM	1.19E-12	**0.64**	0.52
LY96	7.14E-13	**0.64**	0.29
MAFB	2.38E-07	**0.60**	0.24
MAGI1	2.06E-07	**0.60**	-0.27
MAML3	2.38E-08	**0.61**	-0.32
MAOA	3.20E-08	**0.61**	-0.37
MAP3K5	2.46E-10	**0.62**	-0.23
MAP7	2.64E-09	**0.62**	-0.25
MCCC1	3.65E-08	**0.61**	-0.18
ME1	7.25E-10	**0.62**	0.28
MELK	3.41E-07	**0.60**	0.24
MFAP3L	2.94E-11	**0.63**	-0.41
MFAP5	2.57E-10	**0.62**	0.45
MFGE8	6.06E-08	**0.61**	0.19
MGST2	4.17E-09	**0.61**	-0.24
MMD	8.27E-14	**0.64**	0.32
MME	3.65E-07	**0.60**	0.32
MMEL1	2.05E-07	**0.60**	-0.28
MMP11	2.06E-09	**0.62**	0.45
MNDA	1.09E-07	**0.60**	0.30
MOXD1	1.65E-10	**0.62**	0.31
MPP1	5.48E-11	**0.63**	0.28
MRAS	1.27E-07	**0.60**	0.24
MS4A6A	1.23E-08	**0.61**	0.24
MSC	4.33E-10	**0.62**	0.29
MSN	1.29E-09	**0.62**	0.34
MSRB3	3.64E-08	**0.61**	0.26

MSX2	6.76E-09	**0.61**	-0.39
MT1E	8.05E-08	**0.61**	0.27
MT1G	3.93E-13	**0.64**	0.44
MT1X	6.56E-15	**0.65**	0.47
MT2A	2.38E-22	**0.69**	0.62
MTHFD2	2.01E-11	**0.63**	0.32
MYADM	9.81E-10	**0.62**	0.29
MYL9	5.13E-08	**0.61**	0.32
MYLK	9.10E-09	**0.61**	0.36
MYO5A	2.70E-10	**0.62**	0.25
MYO6	2.07E-07	**0.60**	-0.19
MZF1	6.37E-09	**0.61**	-0.14
N6AMT1	3.33E-07	**0.60**	-0.13
NADSYN1	1.61E-11	**0.63**	-0.29
NEDD4L	6.25E-10	**0.62**	-0.29
NEK6	1.71E-10	**0.62**	0.24
NEXN	1.80E-07	**0.60**	0.24
NFIL3	1.29E-09	**0.62**	0.23
NNMT	6.76E-15	**0.65**	0.41
NOD2	1.73E-07	**0.60**	0.28
NPAS2	2.15E-12	**0.64**	-0.35
NPC1	1.93E-10	**0.62**	0.20
NRM	3.23E-08	**0.61**	0.18
NSUN4	6.06E-14	**0.65**	-0.16
NSUN6	1.93E-10	**0.62**	-0.30
NT5DC3	5.53E-09	**0.61**	0.18
NUPR1	2.13E-08	**0.61**	0.30
OFD1	2.03E-09	**0.62**	-0.17
OLFML2B	4.23E-13	**0.64**	0.31
OLR1	2.23E-07	**0.60**	0.33
ORMDL3	9.58E-08	**0.60**	-0.14
OSBPL2	1.21E-08	**0.61**	-0.14
OSMR	4.33E-10	**0.62**	0.41
OSR2	9.58E-08	**0.60**	0.27
P2RY6	2.03E-07	**0.60**	0.22
PACRG	1.15E-08	**0.61**	-0.34
PALLD	1.55E-12	**0.64**	0.40
PAPSS2	9.50E-12	**0.63**	0.28
PAQR8	1.70E-07	**0.60**	-0.27
PARVB	1.66E-07	**0.60**	0.20
PATZ1	9.40E-09	**0.61**	-0.29
PCBD1	9.54E-08	**0.60**	-0.19
PCSK5	7.46E-08	**0.61**	0.23
PDE10A	2.99E-09	**0.62**	-0.41
PDGFC	9.70E-08	**0.60**	0.33
PDGFRB	1.54E-08	**0.61**	0.23
PDLIM3	9.46E-12	**0.63**	0.39
PDPN	1.23E-09	**0.62**	0.26
PGAP1	7.50E-08	**0.61**	-0.25
PGM2L1	1.55E-10	**0.62**	0.25

PGPEP1	1.59E-10	**0.62**	-0.30
PHF19	1.22E-07	**0.60**	0.17
PIGZ	1.22E-08	**0.61**	-0.27
PIK3AP1	1.66E-07	**0.60**	0.26
PIK3C2B	4.90E-08	**0.61**	-0.24
PLA2G2F	2.08E-08	**0.61**	-0.50
PLA2G7	3.05E-07	**0.60**	0.25
PLAU	5.12E-09	**0.61**	0.34
PLAUR	6.30E-10	**0.62**	0.25
PLCD3	8.97E-11	**0.63**	-0.31
PLEK	5.59E-08	**0.61**	0.24
PLEKHA4	4.93E-08	**0.61**	0.26
PLEKHA6	2.72E-11	**0.63**	-0.36
PLEKHA7	7.09E-15	**0.65**	-0.31
PLEKHH1	1.69E-13	**0.64**	-0.36
PLEKHH3	5.96E-10	**0.62**	-0.18
PLS3	1.61E-07	**0.60**	0.21
PLXNB1	4.53E-09	**0.61**	-0.21
PMP22	1.53E-07	**0.60**	0.28
POF1B	1.25E-09	**0.62**	-0.44
POSTN	2.40E-16	**0.66**	0.74
PPARG	5.63E-09	**0.61**	-0.45
PPFIBP2	1.74E-16	**0.66**	-0.46
PPP1R3C	6.07E-09	**0.61**	-0.40
PRDM1	1.15E-07	**0.60**	0.20
PRICKLE1	6.89E-08	**0.61**	0.21
PRKCD	7.94E-08	**0.61**	-0.17
PROM2	1.80E-08	**0.61**	-0.35
PRRX1	3.96E-17	**0.66**	0.50
PTGIS	5.81E-09	**0.61**	0.42
PTGS1	1.11E-08	**0.61**	0.37
PTK6	6.20E-08	**0.61**	-0.24
PTMS	7.15E-08	**0.61**	0.20
PTPRU	1.85E-09	**0.62**	-0.27
RAB11A	2.88E-07	**0.60**	-0.18
RAB15	4.80E-11	**0.63**	-0.33
RAB23	5.37E-11	**0.63**	0.25
RAB3IL1	1.02E-09	**0.62**	0.17
RAI1	2.99E-12	**0.64**	-0.23
RAP1GAP	1.03E-08	**0.61**	-0.29
RAPGEFL1	1.93E-10	**0.62**	-0.36
RARRES2	1.74E-09	**0.62**	0.26
RBP1	5.88E-11	**0.63**	0.38
RCN3	4.39E-12	**0.63**	0.33
RGS1	3.72E-09	**0.62**	0.35
RGS10	1.28E-07	**0.60**	0.20
RGS2	1.84E-12	**0.64**	0.39
RGS4	2.13E-08	**0.61**	0.32
RNASE6	9.54E-08	**0.60**	0.30
RNASEL	1.39E-07	**0.60**	-0.14

RNF128	5.84E-10	**0.62**	-0.48
RNF44	1.45E-07	**0.60**	-0.13
ROR2	1.61E-12	**0.64**	0.41
RXRA	8.49E-09	**0.61**	-0.22
S100A10	1.40E-13	**0.64**	0.47
S100A8	3.75E-09	**0.62**	0.72
SACS	4.01E-09	**0.61**	0.29
SALL4	2.42E-07	**0.60**	0.27
SCAP	1.47E-08	**0.61**	-0.22
SEMA3A	1.18E-07	**0.60**	0.23
SEMA6A	5.14E-08	**0.61**	-0.40
SERPINE2	3.12E-13	**0.64**	0.41
SERPINF1	3.22E-10	**0.62**	0.33
SFRP2	4.70E-14	**0.65**	0.70
SFRP4	5.17E-11	**0.63**	0.56
SGCE	9.95E-08	**0.60**	0.29
SGPP1	9.36E-08	**0.60**	0.31
SH2D4A	5.43E-11	**0.63**	-0.26
SH3YL1	5.41E-09	**0.61**	-0.26
SIRPA	2.75E-09	**0.62**	0.23
SLC14A1	2.39E-11	**0.63**	-0.73
SLC16A10	2.37E-07	**0.60**	0.16
SLC22A5	1.64E-09	**0.62**	-0.21
SLC23A2	5.50E-09	**0.61**	-0.24
SLC24A1	3.45E-10	**0.62**	-0.18
SLC29A3	1.41E-09	**0.62**	-0.30
SLC2A3	6.86E-09	**0.61**	0.31
SLC39A14	6.34E-09	**0.61**	0.21
SLC41A2	2.65E-08	**0.61**	0.23
SLC43A3	5.80E-10	**0.62**	0.28
SLC7A7	2.16E-08	**0.61**	0.21
SLC9A1	2.23E-07	**0.60**	-0.23
SLFN11	1.62E-10	**0.62**	0.30
SLITRK6	2.63E-10	**0.62**	-0.61
SLTM	1.62E-08	**0.61**	-0.15
SMAD3	2.39E-07	**0.60**	-0.22
SMAD6	5.44E-14	**0.65**	-0.39
SNCG	2.33E-07	**0.60**	-0.43
SNX10	3.17E-07	**0.60**	0.24
SOCS3	4.08E-08	**0.61**	0.24
SOD2	2.14E-09	**0.62**	0.26
SORL1	3.07E-10	**0.62**	-0.37
SPATA20	8.00E-10	**0.62**	-0.21
SPHK1	4.23E-13	**0.64**	0.40
SPOCD1	2.06E-07	**0.60**	-0.37
SPON2	1.93E-07	**0.60**	0.29
SPRED2	1.56E-07	**0.60**	-0.17
SRGN	6.82E-09	**0.61**	0.32
SSH3	7.81E-11	**0.63**	-0.31
ST3GAL1	2.07E-09	**0.62**	-0.28

ST3GAL4	9.19E-11	**0.63**	-0.31
ST3GAL5	1.56E-18	**0.67**	-0.46
STAT6	1.95E-07	**0.60**	-0.15
STOM	1.71E-07	**0.60**	0.28
STX2	1.03E-09	**0.62**	0.22
SULF1	1.56E-18	**0.67**	0.60
SULF2	1.05E-11	**0.63**	0.34
SUOX	2.42E-07	**0.60**	-0.18
SYT11	2.57E-07	**0.60**	0.16
SYT17	7.01E-08	**0.61**	-0.30
TAGLN	1.82E-09	**0.62**	0.44
TBX3	2.22E-12	**0.64**	-0.45
TCF4	2.42E-07	**0.60**	0.22
TEAD2	3.70E-10	**0.62**	0.29
TEAD4	7.70E-08	**0.61**	0.18
TESC	1.78E-09	**0.62**	-0.48
TGFB3	3.22E-10	**0.62**	0.29
TGFBI	2.57E-12	**0.64**	0.45
TGM2	5.26E-09	**0.61**	0.33
THBS1	2.38E-07	**0.60**	0.31
THBS2	2.82E-12	**0.64**	0.47
THY1	2.19E-07	**0.60**	0.25
TIMP2	6.45E-14	**0.65**	0.45
TMC7	2.86E-11	**0.63**	-0.34
TMEM154	5.11E-10	**0.62**	-0.31
TMEM158	9.40E-10	**0.62**	0.29
TMEM45A	9.39E-10	**0.62**	0.47
TMEM51	5.25E-09	**0.61**	-0.21
TMEM63A	9.57E-10	**0.62**	-0.21
TNC	6.56E-09	**0.61**	0.36
TNFAIP6	6.69E-14	**0.65**	0.43
TNFRSF12A	8.17E-08	**0.61**	0.26
TNFRSF21	2.78E-07	**0.60**	-0.24
TPM1	4.80E-11	**0.63**	0.32
TPM2	1.70E-10	**0.62**	0.39
TPST1	1.08E-07	**0.60**	0.26
TRAK1	2.91E-12	**0.64**	-0.34
TRERF1	7.70E-08	**0.61**	-0.18
TRIM2	2.03E-09	**0.62**	-0.28
TSEN2	1.07E-07	**0.60**	-0.17
TSPAN14	1.11E-07	**0.60**	-0.17
TSPAN31	1.56E-08	**0.61**	-0.14
TUBB6	3.23E-15	**0.65**	0.39
TWIST1	3.21E-07	**0.60**	0.20
TXNIP	1.04E-09	**0.62**	-0.29
TXNRD1	8.60E-12	**0.63**	0.27
TYROBP	3.23E-08	**0.61**	0.28
UCHL1	6.56E-09	**0.61**	0.40
USP4	1.72E-08	**0.61**	-0.12
VASN	3.74E-07	**0.60**	0.20

VCAN	5.54E-12	**0.63**	0.44
VIM	5.91E-08	**0.61**	0.29
VIPR1	3.85E-08	**0.61**	-0.27
VSIG4	7.72E-08	**0.61**	0.32
WDR6	3.03E-07	**0.60**	-0.19
ZADH2	9.85E-08	**0.60**	-0.17
ZMYND8	1.90E-07	**0.60**	-0.20
ZNF211	7.70E-08	**0.61**	-0.19
ZNF320	1.39E-08	**0.61**	-0.23
ZNF446	1.07E-08	**0.61**	-0.12
ZNF552	5.93E-08	**0.61**	-0.29
ZNF559	2.71E-07	**0.60**	-0.13
ZNF561	9.43E-10	**0.62**	-0.22
ZNF562	1.80E-07	**0.60**	-0.20
ZNF564	1.82E-09	**0.62**	-0.12
ZNF626	5.58E-08	**0.61**	-0.24
ZNF792	6.96E-11	**0.63**	-0.19
ZSCAN16	2.98E-07	**0.60**	-0.19
