# Supplementary material for: Repurposing Amiodarone for Bladder Cancer Treatment
Source: Cancer Res Commun. 2025 Jun 4;5(6):906–20. doi: 10.1158/2767-9764.CRC-24-0433 (PMC12134865; doi:10.1158/2767-9764.CRC-24-0433)
Supplement: Supplementary Table 2 — List of differentially abundant proteins between muscle invasive bladder cancer (MIBC) and non-muscle invasive bladder cancer (NMIBC) [file crc-24-0433_supplementary_table_2_suppst2.pdf]

Supplementary Table 2. List of differentially abundant proteins between muscle invasive bladder cancer (MIBC) and non-muscle invasive bladder cancer (NMIBC)

**UniprotID**	**Symbol**	Proteome Discoverer (high+medium+low confidence)		Proteome Discoverer (high confidence)		Max Quant	
		BH p-value	fold change (Avg. abundance MIBC/ Avg. abundance NMIBC)	BH p-value	fold change (Avg. abundance MIBC/ Avg. abundance NMIBC)	BH p-value	fold change (Avg. abundance MIBC/ Avg. abundance NMIBC)
P0C874	SPATA31D3	3.13E-07	15.83				
P19013	KRT4	4.86E-07	3.36	7.70E-03	2.09	1.66E-03	2.38
Q9C093	SPEF2	2.01E-05	14.45				
O75122	CLASP2	3.85E-05	49.34				
A6NMD2	GOLGA8J	5.92E-05	12.30				
P08729	KRT7	1.27E-04	0.16	6.87E-05	0.16	2.71E-03	0.13
P04179	SOD2	1.28E-04	4.73	1.22E-04	4.54	6.74E-04	3.36
Q96Q05	TRAPPC9	1.88E-04	7.89				
Q969H8	MYDGF	1.92E-04	8.43	1.22E-04	8.83	1.70E-03	6.94
Q96FQ6	S100A16	1.92E-04	10.97	2.25E-04	10.76	4.47E-03	9.50
Q53H82	LACTB2	1.95E-04	5.59				
P35556	FBN2	2.25E-04	0.22				
Q8N3S3	PHTF2	2.25E-04	5.24				
Q8IWB4	SPATA31A7	2.73E-04	6.54				
Q92558	WASF1	2.83E-04	0.10				
Q9NY64	SLC2A8	2.86E-04	13.74				
Q9BY41	HDAC8	2.95E-04	0.21				
P26038	MSN	2.95E-04	5.24	1.60E-04	5.14	4.93E-04	4.03
Q05586	GRIN1	2.95E-04	4.76				
Q08554	DSC1	3.07E-04	0.03				
Q8NBS9	TXNDC5	3.59E-04	3.29	7.24E-04	3.37	1.21E-02	2.84
Q9UHI8	ADAMTS1	3.60E-04	3.09				
Q5VWG9	TAF3	3.60E-04	7.57				
Q9BRF8	CPPED1	3.60E-04	8.06			2.79E-03	6.40
Q5JV73	FRMPD3	3.78E-04	8.08				
O43559	FRS3	4.30E-04	0.09				
Q6Y7W6	GIGYF2	4.38E-04	4.69				
Q9HCB6	SPON1	4.46E-04	0.06				
Q5T013	HYI	5.51E-04	11.30	8.31E-03	8.93		
C9J069	AJM1	5.89E-04	0.22				
Q9UL45	BLOC1S6	6.25E-04	11.64				
Q8WXH2	JPH3	6.40E-04	0.03				
Q9GZT5	WNT10A	6.40E-04	0.17				
Q9Y4W6	AFG3L2	6.74E-04	4.51	1.77E-02	3.07		
Q14003	KCNC3	6.74E-04	10.56				
Q6ZU69	FAM205A	6.74E-04	8.09				
Q6NX49	ZNF544	6.84E-04	0.00				
Q9UM11	FZR1	6.84E-04	0.00				
Q9P2E9	RRBP1	6.84E-04	3.85	1.13E-03	3.33	2.79E-03	4.05
Q08722	CD47	6.84E-04	3.19	1.90E-04	3.51	2.03E-03	5.15
Q9C0J9	BHLHE41	6.90E-04	0.15				
Q9NP79	VTA1	7.31E-04	0.13				
P19971	TYMP	7.66E-04	6.88	3.81E-04	6.90	8.36E-03	7.44
Q5T1M5	FKBP15	7.66E-04	9.87				
Q8N3Z0	PRSS35	7.66E-04	4.80				
A6NNV3	SPDYE16	7.66E-04	5.91				
Q6ZS86	GK5	7.66E-04	5.97				
O00410	IPO5	8.64E-04	6.27	8.47E-03	5.35		
Q8NCA5	FAM98A	1.08E-03	7.53				
Q9UBR2	CTSZ	1.09E-03	3.05	5.92E-04	3.14	3.57E-02	2.84
B5MCY1	TDRD15	1.12E-03	14.38				
P45452	MMP13	1.12E-03	9.41				

Q86UA1	PRPF39	1.26E-03	0.14				
Q15042	RAB3GAP1	1.29E-03	10.17				
Q6BDS2	UHRF1BP1	1.30E-03	2.12				
Q7Z5K2	WAPL	1.30E-03	2.81				
P12429	ANXA3	1.48E-03	7.41	7.24E-04	7.89	9.68E-06	10.86
Q9BUB7	TMEM70	1.48E-03	8.27				
Q9Y6H5	SNCAIP	1.59E-03	0.10				
O76090	BEST1	1.65E-03	0.03	3.81E-04	0.00		
Q9Y2H6	FNDC3A	1.73E-03	8.91				
Q8TE77	SSH3	1.79E-03	0.27	1.08E-03	0.26	8.26E-04	0.13
Q96BZ4	PLD4	1.79E-03	0.21				
O75069	TMCC2	1.79E-03	0.29				
Q6ZMZ0	RNF19B	1.79E-03	0.29				
P41091	EIF2S3	1.79E-03	3.95	4.50E-04	4.11	1.66E-03	5.09
Q9Y617	PSAT1	1.79E-03	9.06	1.08E-03	9.15		
Q8WWK9	CKAP2	1.79E-03	7.97				
Q86XP3	DDX42	1.79E-03	3.68				
Q9Y6Q3	ZFP37	1.79E-03	8.15				
P15822	HIVEP1	1.79E-03	4.42				
Q68EM7	ARHGAP17	1.79E-03	0.26				
Q15006	EMC2	1.79E-03	9.10	1.07E-03	9.47	2.77E-04	15.05
O43447	PPIH	1.79E-03	3.45	1.29E-02	2.97		
A8MW95	BECN2	1.79E-03	only in MIBC				
Q8WXS3	BAALC	1.79E-03	only in MIBC				
Q92793	CREBBP	1.80E-03	0.16				
Q9Y3A5	SBDS	1.83E-03	4.53	1.11E-03	4.55	2.90E-02	1.02
Q9HA38	ZMAT3	1.83E-03	3.47				
Q9NZ71	RTEL1	1.85E-03	4.62				
Q9UBF9	MYOT	1.85E-03	4.08				
P30533	LRPAP1	1.88E-03	3.03				
Q9H1P3	OSBPL2	1.89E-03	0.24	1.79E-03	0.25		
O95197	RTN3	1.98E-03	2.88	2.81E-03	2.53	3.53E-02	4.99
Q2M2D7	TBC1D28	2.22E-03	0.26				
Q05315	CLC	2.34E-03	4.97	1.46E-03	4.86		
Q9P0X4	CACNA1I	2.34E-03	5.77				
Q9Y696	CLIC4	2.36E-03	3.20	1.86E-05	6.66	5.49E-04	6.86
Q13148	TARDBP	2.51E-03	0.40				
Q9UBS4	DNAJB11	2.51E-03	8.30	1.82E-04	11.26	8.26E-04	27.97
Q9BYK8	HELZ2	2.51E-03	4.97				
Q4G0Z9	MCMDC2	2.54E-03	0.13				
Q07065	CKAP4	2.57E-03	2.73	1.46E-03	2.33	1.78E-02	1.96
Q9H9F9	ACTR5	2.62E-03	15.81				
O75419	CDC45	2.75E-03	0.07				
Q8TC41	RNF217	2.75E-03	2.98				
P21506	ZNF10	2.75E-03	2.35				
Q8NHX9	TPCN2	2.76E-03	5.81				
P55809	OXCT1	2.84E-03	10.91	1.83E-03	12.18	5.62E-03	9.79
Q8IYW2	CFAP46	2.84E-03	3.94				
Q5VTL8	PRPF38B	2.90E-03	0.12				
O75879	GATB	2.94E-03	2.81				
P06702	S100A9	3.00E-03	5.09	1.87E-03	5.09	5.53E-03	4.10
Q96K30	RITA1	3.00E-03	0.16				
Q6ZNE9	RUFY4	3.12E-03	0.29				
Q71U36	TUBA1A	3.22E-03	0.55	1.59E-03	0.55		
O43361	ZNF749	3.24E-03	0.16				
Q5JWF2	GNAS	3.30E-03	0.03	2.28E-03	0.00		
P49321	NASP	3.48E-03	2.54	1.70E-03	2.61	1.35E-02	1.87
Q6IV72	ZNF425	3.49E-03	5.74				
H7BZ55	CROCC2	3.51E-03	0.24				
P49788	RARRES1	3.51E-03	4.77	1.08E-03	4.60	1.36E-02	3.94
P15104	GLUL	3.51E-03	10.54	8.81E-04	29.87	7.22E-06	16.30

Q9Y5H7	PCDHA5	3.51E-03	0.06				
Q9GZM7	TINAGL1	3.69E-03	0.16	2.13E-03	0.15	7.29E-03	0.14
P53618	COPB1	3.69E-03	2.86	2.21E-03	2.99	7.08E-03	2.67
Q9H3N8	HRH4	3.69E-03	11.49				
P13284	IFI30	3.81E-03	1.58	2.66E-06	14.97		
Q86V88	MDP1	3.81E-03	5.86				
P51617	IRAK1	3.88E-03	0.30				
B2RTY4	MYO9A	4.16E-03	3.71	1.59E-05	9.69		
Q9H4G4	GLIPR2	4.16E-03	9.12	2.70E-03	9.15	2.79E-03	10.24
Q96N46	TTC14	4.24E-03	0.23				
Q8NEE8	TTC16	4.24E-03	6.90				
Q86TB9	PATL1	4.31E-03	0.11				
Q9H2X9	SLC12A5	4.31E-03	5.68				
Q92905	COPS5	4.31E-03	0.06	2.81E-03	0.06	3.53E-02	0.06
O00750	PIK3C2B	4.46E-03	4.68				
P62857	RPS28	4.58E-03	0.19	3.03E-03	0.19		
Q9Y232	CDYL	4.58E-03	2.32				
Q8NEM7	SUPT20H	4.72E-03	7.42				
P59665	DEFA1	4.86E-03	4.71	3.01E-03	4.78		
Q6F5E8	CARMIL2	4.86E-03	3.66				
O14745	SLC9A3R1	4.93E-03	9.71	2.03E-04	17.47	2.33E-04	36.42
Q96FK6	WDR89	5.01E-03	6.57				
P15428	HPGD	5.02E-03	0.59	3.34E-03	0.57	3.84E-03	0.56
Q9UIW2	PLXNA1	5.04E-03	3.64				
Q9BZF3	OSBPL6	5.24E-03	23.99				
Q92562	FIG4	5.35E-03	0.38				
P14625	HSP90B1	5.35E-03	1.73	2.87E-03	1.72	5.14E-03	1.85
Q9BYV6	TRIM55	5.35E-03	2.50				
P20794	MAK	5.35E-03	4.30				
Q86WN1	FCHSD1	5.35E-03	4.09				
Q9P2E5	CHPF2	5.35E-03	117.93				
Q9H0U3	MAGT1	5.43E-03	2.50			1.09E-02	4.53
Q9UIS9	MBD1	5.48E-03	0.26				
P02751	FN1	5.48E-03	3.93	4.13E-03	3.92	2.79E-03	4.64
Q17RR3	PNLIPRP3	5.48E-03	3.21				
Q3SYG4	BBS9	5.49E-03	0.39	2.18E-03	0.38		
P05109	S100A8	5.61E-03	6.39	4.02E-03	6.59	1.35E-02	7.65
P49915	GMPS	5.61E-03	2.19				
Q6PJ61	FBXO46	5.61E-03	7.82				
Q6U841	SLC4A10	5.73E-03	2.81				
P36776	LONP1	5.75E-03	2.85	2.24E-03	3.14	1.30E-02	2.55
Q9UC06	ZNF70	6.02E-03	0.32				
Q9H8M2	BRD9	6.19E-03	2.60				
P55145	MANF	6.27E-03	6.46	4.02E-03	6.75		
O60240	PLIN1	6.34E-03	2.39				
P07099	EPHX1	6.50E-03	3.01	4.02E-03	3.06	2.32E-02	3.26
Q13162	PRDX4	6.53E-03	2.00	4.76E-03	2.03	1.00E-05	9.08
Q9BV40	VAMP8	6.53E-03	2.74	2.00E-03	2.77		
Q00059	TFAM	6.56E-03	3.76	3.50E-02	3.58		
Q9UHB9	SRP68	6.65E-03	2.90	4.02E-03	5.31		
Q03013	GSTM4	6.67E-03	0.16				
P25685	DNAJB1	6.75E-03	2.75	4.51E-03	3.17		
Q9Y520	PRRC2C	6.75E-03	3.35				
O15050	TRANK1	6.82E-03	0.33				
P42765	ACAA2	6.82E-03	3.62	2.87E-03	3.71	4.02E-02	1.33
Q14232	EIF2B1	6.82E-03	4.38	4.54E-03	4.42		
Q14554	PDIA5	6.84E-03	0.13				
Q8NE01	CNNM3	6.84E-03	0.12				
O75064	DENND4B	6.84E-03	8.44				
P42766	RPL35	6.85E-03	1.73	4.76E-03	1.72		
Q1A5X6	IQCJ	6.86E-03	6.63				

P15153	RAC2	6.88E-03	8.25	2.75E-02	14.28	2.89E-02	21.69
P09874	PARP1	6.95E-03	2.22	8.33E-03	2.26		
Q6ZRI6	C15orf39	7.22E-03	36.77				
Q9P0M6	MACROH2A2	7.25E-03	0.48	4.40E-03	0.49	9.81E-03	0.49
P05091	ALDH2	7.25E-03	9.46			6.92E-03	7.94
O00203	AP3B1	7.29E-03	2.35				
P25325	MPST	7.32E-03	1.94	4.76E-03	1.97		
Q9NXS2	QPCTL	7.32E-03	6.70				
Q15131	CDK10	7.32E-03	7.32				
K9M1U5	IFNL4	7.32E-03	7.81				
P02686	MBP	7.43E-03	1.93				
Q03938	ZNF90	7.45E-03	0.33				
P63173	RPL38	7.50E-03	2.79	5.26E-03	2.80	6.59E-03	3.40
P22612	PRKACG	7.50E-03	5.70	4.76E-03	5.76		
Q14956	GPNMB	7.65E-03	2.57	8.61E-03	2.63		
O95232	LUC7L3	7.71E-03	2.38				
P46531	NOTCH1	7.73E-03	0.21				
P08727	KRT19	7.92E-03	0.40	4.76E-03	0.40	1.11E-02	0.01
Q49MI3	CERKL	7.92E-03	0.20				
P61916	NPC2	7.92E-03	15.25	5.22E-03	17.46		
Q8WVI7	PPP1R1C	8.07E-03	4.28				
Q5T197	DCST1	8.21E-03	0.10				
O43852	CALU	8.21E-03	3.23	5.29E-03	3.15	3.44E-04	4.12
Q9Y6Q1	CAPN6	8.51E-03	3.95				
Q96LI6	HSFY1	8.92E-03	8.59				
Q9NP72	RAB18	9.02E-03	3.26	5.63E-03	3.40		
Q5T7W7	TSTD2	9.02E-03	2.46				
Q8TCP9	FAM200A	9.02E-03	0.14				
P0CB33	ZNF735	9.02E-03	5.70				
Q9P0V9	SEPTIN10	9.03E-03	4.50	8.41E-03	4.58		
P05161	ISG15	9.08E-03	3.43	8.33E-03	3.42	1.07E-02	2.45
O43837	IDH3B	9.08E-03	4.72	6.54E-03	4.64		
Q8NFW1	COL22A1	9.34E-03	0.36				
Q8IUE6	H2AC21	9.34E-03	2.75	6.05E-03	2.72		
O75880	SCO1	9.34E-03	4.46	1.60E-04	8.47		
P0DI83	RAB34	9.34E-03	4.05				
P16333	NCK1	9.34E-03	5.89				
O14981	BTAF1	9.34E-03	0.15				
O14639	ABLIM1	9.34E-03	0.16				
Q9GZZ9	UBA5	9.34E-03	3.61	8.99E-04	11.73		
P30414	NKTR	9.46E-03	0.40				
Q9Y2K3	MYH15	9.47E-03	20.10				
Q96PV6	LENG8	9.66E-03	6.98				
P34931	HSPA1L	9.83E-03	4.09	6.92E-03	4.18		
Q16563	SYPL1	9.93E-03	0.51	6.17E-03	0.50	3.53E-02	0.59
P0DJD1	RGPD2	1.02E-02	3.81				
Q14154	DELE1	1.02E-02	7.07				
A6NMB1	SIGLEC16	1.02E-02	1.67	8.33E-03	1.68		
P03956	MMP1	1.02E-02	2.73				
Q8N302	AGGF1	1.02E-02	2.03				
P22794	EVI2A	1.02E-02	6.39				
Q7L5Y9	MAEA	1.03E-02	3.70				
Q9Y6N5	SQOR	1.04E-02	3.30	6.17E-03	3.32	8.26E-04	5.86
O43633	CHMP2A	1.05E-02	0.12				
O15034	RIMBP2	1.08E-02	0.10				
Q15582	TGFBI	1.08E-02	2.40	8.33E-03	2.29	1.66E-03	2.82
Q96A57	TMEM230	1.11E-02	5.21	7.49E-03	5.39		
Q96J94	PIWIL1	1.13E-02	0.21				
Q8N5C6	SRBD1	1.13E-02	2.92				
A1L3X0	ELOVL7	1.13E-02	8.04				
O60682	MSC	1.13E-02	6.45				

P04035	HMGCR	1.13E-02	5.54				
P13796	LCP1	1.14E-02	3.02	1.47E-02	2.80	7.62E-03	3.17
Q9ULI4	KIF26A	1.15E-02	0.01				
Q5XKE5	KRT79	1.16E-02	0.37	5.52E-03	0.36		
Q96RS0	TGS1	1.16E-02	0.22				
P30740	SERPINB1	1.16E-02	3.45	7.49E-03	3.46	2.98E-02	3.20
Q56VL3	OCIAD2	1.16E-02	3.75				
Q8IXB1	DNAJC10	1.16E-02	1.83	1.08E-03	10.06		
Q92797	SYMPK	1.16E-02	5.07				
A6NCI4	VWA3A	1.16E-02	6.01				
Q2M1P5	KIF7	1.16E-02	13.51				
P42898	MTHFR	1.16E-02	0.27				
O95185	UNC5C	1.16E-02	3.39	8.33E-03	3.51		
P0C7T5	ATXN1L	1.17E-02	0.24				
Q9UKR5	ERG28	1.17E-02	5.93	7.70E-03	6.11		
P11021	HSPA5	1.17E-02	1.64	8.61E-03	1.61	6.23E-03	1.65
P46100	ATRX	1.18E-02	0.31				
Q9NXR1	NDE1	1.20E-02	6.29				
P13535	MYH8	1.25E-02	0.25				
Q6IBS0	TWF2	1.25E-02	0.24	9.20E-03	0.25	1.82E-02	0.22
P07384	CAPN1	1.25E-02	0.52	5.76E-03	0.51	6.21E-03	0.42
P15622	ZNF250	1.25E-02	0.05				
P20849	COL9A1	1.25E-02	0.06				
O15055	PER2	1.25E-02	0.14				
Q14966	ZNF638	1.25E-02	0.21				
Q96SB8	SMC6	1.25E-02	0.38				
Q56UN5	MAP3K19	1.25E-02	0.59				
Q9BX69	CARD6	1.25E-02	0.61				
Q9Y4L1	HYOU1	1.25E-02	1.82	4.76E-03	1.84	1.38E-02	1.76
Q7KZF4	SND1	1.25E-02	2.10	8.33E-03	2.37	6.05E-03	2.82
Q92820	GGH	1.25E-02	4.79	7.49E-03	4.55	7.29E-03	4.40
P26583	HMGB2	1.25E-02	4.08	1.15E-02	4.39	7.54E-03	4.87
O14828	SCAMP3	1.25E-02	3.30	7.49E-03	3.36	1.34E-02	5.88
Q9Y5P6	GMPPB	1.25E-02	3.95				
P49458	SRP9	1.25E-02	only in MIBC				
Q9UK17	KCND3	1.25E-02	3.24	5.63E-03	3.59		
O00233	PSMD9	1.25E-02	4.77				
Q9NRR4	DROSHA	1.25E-02	2.33				
O75426	FBXO24	1.25E-02	1.20				
Q9HBR0	SLC38A10	1.25E-02	2.19				
Q7Z6L0	PRRT2	1.25E-02	2.49				
Q01201	RELB	1.25E-02	4.05				
Q6PIV2	FOXR1	1.25E-02	6.23				
Q13207	TBX2	1.25E-02	9.94				
Q9C0G0	ZNF407	1.25E-02	11.55				
Q99959	PKP2	1.25E-02	23.31				
A2RUS2	DENND3	1.25E-02	0.12				
Q8IWX7	UNC45B	1.25E-02	0.47				
Q9BVJ7	DUSP23	1.25E-02	only in MIBC				
Q9C026	TRIM9	1.25E-02	2.34				
Q9UBK8	MTRR	1.25E-02	3.36				
Q9UJ96	KCNG2	1.25E-02	3.55				
Q6P1Q0	LETMD1	1.25E-02	3.66				
Q96EY5	MVB12A	1.25E-02	4.19				
Q96NU1	SAMD11	1.26E-02	7.66				
Q9NSV4	DIAPH3	1.27E-02	0.28	1.41E-03	0.05		
Q8NA56	TTC29	1.27E-02	0.23				
P32455	GBP1	1.27E-02	12.27	9.20E-03	12.54		
Q69YI7	NAIF1	1.27E-02	4.53				
Q99497	PARK7	1.27E-02	0.58	1.12E-02	0.60	2.86E-04	0.43
Q9HD40	SEPSECS	1.28E-02	5.30				

P20962	PTMS	1.30E-02	2.96	4.76E-03	3.73		
P00846	MT-ATP6	1.30E-02	8.30	8.61E-03	8.65		
P05164	MPO	1.33E-02	5.50	1.56E-02	7.58	1.46E-02	6.41
O60716	CTNND1	1.37E-02	0.38	1.53E-02	0.39	6.92E-03	0.30
Q5T1R4	HIVEP3	1.37E-02	0.38				
Q13356	PPIL2	1.40E-02	2.90				
P08621	SNRNP70	1.42E-02	2.73	8.61E-03	2.81	2.96E-02	2.74
Q96PR1	KCNC2	1.43E-02	0.34				
Q0VF49	KIAA2012	1.43E-02	1.70				
Q13277	STX3	1.43E-02	9.13				
P09211	GSTP1	1.43E-02	0.47	8.61E-03	0.46	4.33E-03	0.40
P14618	PKM	1.43E-02	0.54	8.61E-03	0.54	6.56E-03	0.50
Q15125	EBP	1.45E-02	5.99	9.51E-03	5.96	3.65E-04	11.78
P51948	MNAT1	1.45E-02	0.02				
O15530	PDPK1	1.46E-02	2.76				
Q16181	SEPTIN7	1.47E-02	0.59				
P49588	AARS1	1.51E-02	2.58			3.33E-02	2.17
Q96JN2	CCDC136	1.51E-02	3.57				
P11387	TOP1	1.51E-02	9.21				
Q96QE2	SLC2A13	1.52E-02	26.68				
Q99250	SCN2A	1.53E-02	0.17				
Q7L8L6	FASTKD5	1.53E-02	2.90				
Q13136	PPFIA1	1.53E-02	4.08				
Q6UXB8	PI16	1.53E-02	4.52				
Q96QK1	VPS35	1.55E-02	0.16	9.10E-03	0.16		
P18283	GPX2	1.56E-02	0.16	1.05E-02	0.16	7.29E-03	0.00
P49750	YLPM1	1.56E-02	0.09				
O60218	AKR1B10	1.60E-02	5.68	8.33E-03	5.57		
Q9BVK6	TMED9	1.61E-02	1.59	4.51E-03	1.67	2.18E-02	1.95
Q99680	GPR22	1.61E-02	2.98				
P82673	MRPS35	1.64E-02	0.31				
Q8IWL3	HSCB	1.65E-02	0.06				
P54764	EPHA4	1.66E-02	0.12				
P43686	PSMC4	1.72E-02	2.34	1.30E-02	2.32		
Q9UM54	MYO6	1.73E-02	0.24				
O60566	BUB1B	1.74E-02	0.39				
Q16610	ECM1	1.74E-02	3.68				
P08779	KRT16	1.74E-02	14.67	1.21E-02	14.39		
P15559	NQO1	1.76E-02	0.72	1.27E-02	0.69	4.63E-02	0.59
Q53EL6	PDCD4	1.77E-02	0.23				
P02538	KRT6A	1.77E-02	39.38	1.14E-02	38.37	9.14E-03	52.06
P53992	SEC24C	1.77E-02	4.13				
Q9Y534	CSDC2	1.80E-02	0.25				
P0C7X5	ZNF806	1.81E-02	3.10	2.33E-04	4.45		
O15533	TAPBP	1.81E-02	4.24	1.15E-02	4.37	3.55E-02	7.00
P62873	GNB1	1.82E-02	2.65	1.13E-02	2.63		
P09467	FBP1	1.83E-02	0.39	8.33E-03	0.36	2.13E-02	0.37
P07195	LDHB	1.83E-02	0.59	8.33E-03	0.58	6.59E-03	0.57
Q96RT7	TUBGCP6	1.83E-02	5.53				
Q14683	SMC1A	1.83E-02	2.12				
Q6PI47	KCTD18	1.83E-02	0.00				
Q9UMY1	NOL7	1.83E-02	0.07				
Q5JPB2	ZNF831	1.86E-02	0.44				
Q9BRX8	PRXL2A	1.88E-02	0.16	1.27E-02	0.16	8.94E-03	0.11
Q96HM7	PCED1B	1.89E-02	4.25				
Q9BQG0	MYBBP1A	1.90E-02	2.72				
Q12778	FOXO1	1.92E-02	0.28				
Q92731	ESR2	1.94E-02	0.27				
A6NDA9	LRIT2	1.97E-02	6.78				
O15015	ZNF646	1.98E-02	0.30				
O14974	PPP1R12A	1.98E-02	3.57				

O60268	KIAA0513	1.99E-02	3.33				
Q86UW2	SLC51B	2.02E-02	3.39				
Q06190	PPP2R3A	2.06E-02	0.36				
Q12931	TRAP1	2.06E-02	3.51	1.49E-02	3.64		
P12004	PCNA	2.10E-02	1.82	1.21E-02	1.88	3.08E-02	2.20
Q96N67	DOCK7	2.13E-02	0.42				
Q96P68	OXGR1	2.14E-02	6.62				
Q15005	SPCS2	2.14E-02	3.22	3.42E-02	4.43		
Q86XA9	HEATR5A	2.15E-02	0.00	1.47E-02	0.00		
Q92522	H1-10	2.16E-02	2.05	4.38E-02	2.00	4.35E-02	2.21
P07476	IVL	2.16E-02	0.31	1.29E-02	0.30	1.10E-02	0.20
Q9Y5I2	PCDHA10	2.16E-02	0.12	1.46E-02	0.12		
Q9HB96	FANCE	2.19E-02	0.28				
Q9Y5K3	PCYT1B	2.26E-02	0.28				
P61964	WDR5	2.28E-02	2.98				
O95361	TRIM16	2.32E-02	4.94				
Q03519	TAP2	2.33E-02	7.55			7.83E-05	15.24
Q8WUY3	PRUNE2	2.34E-02	0.25				
P09601	HMOX1	2.34E-02	2.61				
Q495X7	TRIM60	2.36E-02	0.16				
Q96BS2	TESC	2.38E-02	0.02	1.57E-02	0.01		
O60879	DIAPH2	2.38E-02	0.08				
Q8WYA0	IFT81	2.41E-02	0.42				
Q99735	MGST2	2.41E-02	0.58	1.41E-02	0.58		
Q5K651	SAMD9	2.43E-02	4.73				
Q86XN8	MEX3D	2.43E-02	0.05				
Q9ULE3	DENND2A	2.43E-02	0.24				
Q9BRQ6	CHCHD6	2.43E-02	2.02				
Q5JW98	CALHM4	2.43E-02	0.23				
P19012	KRT15	2.44E-02	0.47	1.47E-02	0.46		
P13929	ENO3	2.44E-02	0.63	1.27E-02	0.63		
Q92621	NUP205	2.44E-02	0.31				
Q99501	GAS2L1	2.44E-02	0.59				
Q495T6	MMEL1	2.44E-02	0.04				
O95070	YIF1A	2.44E-02	2.72				
Q9P1Z0	ZBTB4	2.44E-02	2.81				
O60488	ACSL4	2.44E-02	5.18	1.68E-02	5.18		
Q9UPP1	PHF8	2.44E-02	3.93				
O75347	TBCA	2.47E-02	2.76	8.31E-03	3.03	4.33E-03	4.13
Q9P0L0	VAPA	2.49E-02	2.40	2.01E-02	2.43	4.72E-02	2.42
Q460N3	PARP15	2.49E-02	2.46				
O94972	TRIM37	2.50E-02	0.16				
A0A0B4J240	TRAV10	2.50E-02	2.40				
H0Y7S4	PRAMEF26	2.54E-02	4.96				
Q13268	DHRS2	2.55E-02	0.71	1.56E-02	0.69	1.35E-02	0.69
O00625	PIR	2.58E-02	0.10	1.73E-02	0.10		
Q7L985	LINGO2	2.58E-02	0.08				
Q6PIW4	FIGNL1	2.58E-02	0.29				
Q9Y3Z3	SAMHD1	2.58E-02	4.04	4.29E-02	3.33	1.84E-02	3.51
Q9Y3Q4	HCN4	2.59E-02	0.03				
Q05682	CALD1	2.60E-02	1.28	2.78E-02	1.13		
P20292	ALOX5AP	2.61E-02	2.45	2.17E-02	2.40	5.08E-03	19.04
Q09328	MGAT5	2.70E-02	0.42				
P06703	S100A6	2.72E-02	0.53	1.59E-02	0.53	8.12E-03	0.46
Q15847	ADIRF	2.72E-02	0.74	1.59E-02	0.75	8.36E-03	0.59
Q6NUP7	PPP4R4	2.72E-02	0.24				
Q08334	IL10RB	2.72E-02	0.29				
P54132	BLM	2.73E-02	2.78	6.72E-04	4.35		
Q16851	UGP2	2.73E-02	1.88	1.00E-02	1.97		
P09913	IFIT2	2.74E-02	0.09				
P24821	TNC	2.74E-02	2.85	2.74E-02	3.61	5.49E-04	22.20

P12035	KRT3	2.74E-02	2.88	1.50E-04	3.68		
Q15181	PPA1	2.75E-02	0.49	1.98E-02	0.50		
P00352	ALDH1A1	2.75E-02	7.55			6.92E-03	7.07
P15941	MUC1	2.75E-02	4.43	1.77E-02	4.28	3.53E-02	11.14
Q9P203	BTBD7	2.76E-02	3.19				
Q9H255	OR51E2	2.76E-02	3.49				
Q9UKY7	CDV3	2.81E-02	0.37				
Q9P2E3	ZNFX1	2.81E-02	0.54				
O43516	WIPF1	2.82E-02	0.00				
Q04637	EIF4G1	2.83E-02	1.96	3.72E-03	2.12		
P53621	COPA	2.86E-02	2.39	2.25E-02	2.41	1.45E-02	3.25
P50613	CDK7	2.87E-02	3.23				
P55265	ADAR	2.89E-02	3.13			2.79E-02	2.85
Q96HB5	CCDC120	2.91E-02	2.02				
Q9Y5Z4	HEBP2	2.93E-02	0.49	2.55E-02	0.48		
Q9BRP8	PYM1	2.93E-02	0.35	2.82E-02	0.36		
Q5THK1	PRR14L	2.93E-02	0.50				
P35869	AHR	2.93E-02	1.76				
Q9HCJ6	VAT1L	2.94E-02	2.43				
O60506	SYNCRIP	2.94E-02	0.45	1.54E-02	0.43	1.09E-02	0.41
P87889	ERVK-10	2.96E-02	0.07				
Q16881	TXNRD1	2.97E-02	12.82				
P26358	DNMT1	2.97E-02	3.21				
Q9BU64	CENPO	2.97E-02	1.55				
P27694	RPA1	3.00E-02	3.89				
Q15021	NCAPD2	3.01E-02	2.74	4.76E-03	11.74		
Q7Z3D6	DGLUCY	3.01E-02	0.16				
Q69YN4	VIRMA	3.02E-02	0.31				
Q9NR31	SAR1A	3.03E-02	1.79	2.04E-02	1.79		
P17612	PRKACA	3.04E-02	3.36	1.30E-02	3.48		
P18827	SDC1	3.04E-02	0.46	2.01E-02	0.44	4.25E-02	0.51
Q70EK9	USP51	3.04E-02	2.55				
Q13416	ORC2	3.06E-02	4.83				
Q9Y328	NSG2	3.06E-02	6.26				
D6RIA3	C4orf54	3.08E-02	0.32				
P18583	SON	3.16E-02	0.19				
Q9ULK2	ATXN7L1	3.16E-02	2.91	1.68E-06	only in MIBC		
Q9Y6Q5	AP1M2	3.23E-02	0.00	2.22E-02	0.00		
Q9Y6S9	RPS6KL1	3.23E-02	0.28				
Q04917	YWHAH	3.25E-02	1.70	2.76E-02	1.71		
Q6ZMI0	PPP1R21	3.27E-02	3.23				
Q9Y4G6	TLN2	3.28E-02	1.83				
P26641	EEF1G	3.28E-02	1.75	1.96E-02	1.76	5.53E-03	2.50
P11279	LAMP1	3.28E-02	1.54	2.00E-02	1.56		
Q9Y490	TLN1	3.32E-02	1.62	3.79E-02	1.46	1.85E-02	1.63
A4D161	FAM221A	3.32E-02	3.46				
Q9NPE2	NGRN	3.36E-02	9.00				
B4DU55	ZNF879	3.37E-02	6.02				
Q8N2S1	LTBP4	3.37E-02	4.58	3.45E-02	6.67		
Q53R12	TM4SF20	3.38E-02	0.05				
P51114	FXR1	3.45E-02	2.69	1.95E-02	2.82	3.47E-02	3.63
O75093	SLIT1	3.48E-02	0.51				
P56134	ATP5MF	3.49E-02	0.43	2.56E-02	0.44		
Q14573	ITPR3	3.51E-02	7.45				
O14908	GIPC1	3.51E-02	3.27	2.51E-02	3.19		
O94941	UBOX5	3.52E-02	0.11	2.83E-02	0.04		
Q8NF91	SYNE1	3.52E-02	1.41				
P22392	NME2	3.53E-02	0.49	2.55E-02	0.48		
A0A1B0GVG6	TEX54	3.54E-02	4.60				
Q66K79	CPZ	3.60E-02	0.02				
P47895	ALDH1A3	3.62E-02	4.21	8.33E-03	7.86		

P15538	CYP11B1	3.62E-02	2.13				
P50995	ANXA11	3.62E-02	0.55	1.95E-02	0.54		
Q99590	SCAF11	3.68E-02	1.94				
O00519	FAAH	3.68E-02	0.19				
Q9BW71	HIRIP3	3.70E-02	0.37				
Q14CX7	NAA25	3.75E-02	1.16				
Q9UBQ7	GRHPR	3.77E-02	2.68				
Q5T5U3	ARHGAP21	3.77E-02	7.75				
Q5KSL6	DGKK	3.77E-02	2.28				
A0A1B0GUT2	C10orf143	3.77E-02	0.04				
P63220	RPS21	3.82E-02	0.07	2.54E-02	0.07		
Q96B18	DACT3	3.82E-02	3.79				
P49910	ZNF165	3.83E-02	1.85				
Q4G148	GXYLT1	3.96E-02	0.55				
Q13228	SELENBP1	3.96E-02	6.26	3.02E-02	6.30	2.17E-02	9.28
Q9HCY8	S100A14	3.96E-02	14.25	2.08E-02	14.20	2.47E-02	14.24
Q96ST3	SIN3A	3.96E-02	1.66				
P51449	RORC	3.96E-02	3.03				
Q8TDN4	CABLES1	3.96E-02	3.79				
Q9H1Z9	TSPAN10	4.00E-02	0.25				
P25815	S100P	4.01E-02	0.50	2.47E-02	0.49	9.94E-03	0.48
Q15629	TRAM1	4.02E-02	3.84	2.90E-02	3.83		
Q9UJ72	ANXA10	4.04E-02	0.18	2.57E-02	0.17	1.35E-02	0.14
P55199	ELL	4.04E-02	0.21				
Q56UQ5	N/A	4.06E-02	4.35	2.69E-02	4.33		
Q9NR56	MBNL1	4.08E-02	0.12	4.32E-02	0.12		
P16885	PLCG2	4.08E-02	0.18				
Q9NX63	CHCHD3	4.08E-02	2.41	2.75E-02	2.48		
P80294	MT1H	4.08E-02	3.18				
O60861	GAS7	4.09E-02	5.04				
Q96P26	NT5C1B	4.10E-02	2.20				
O60493	SNX3	4.12E-02	2.37	2.91E-02	2.31		
Q8TBY8	PMFBP1	4.16E-02	0.55				
O75197	LRP5	4.19E-02	0.00	3.15E-02	0.00		
Q96MY1	NOL4L	4.19E-02	0.00				
Q8NDP4	ZNF439	4.19E-02	0.00				
Q9HCS2	CYP4F12	4.19E-02	0.03				
P43490	NAMPT	4.21E-02	2.02				
Q9NPJ3	ACOT13	4.23E-02	5.03	2.59E-02	5.28		
O60784	TOM1	4.23E-02	14.30				
P42330	AKR1C3	4.26E-02	0.46	2.79E-02	0.45		
Q2M1Z3	ARHGAP31	4.26E-02	0.17				
Q93008	USP9X	4.27E-02	1.98	1.74E-02	3.69		
P35611	ADD1	4.29E-02	0.09			9.14E-03	0.27
Q02040	AKAP17A	4.30E-02	0.31				
Q9Y678	COPG1	4.33E-02	2.22	8.95E-03	2.80		
Q09428	ABCC8	4.33E-02	3.80				
Q6UXY8	TMC5	4.33E-02	0.03				
Q9UKE5	TNIK	4.34E-02	1.88				
Q6ZRC1	C4orf50	4.34E-02	0.33				
Q9NXL9	MCM9	4.36E-02	0.03				
Q9BTC0	DIDO1	4.41E-02	1.72				
Q9H223	EHD4	4.44E-02	0.47	2.18E-02	0.44	1.45E-02	0.44
Q9UPN7	PPP6R1	4.46E-02	3.69				
Q8N9B5	JMY	4.47E-02	1.54				
O43623	SNAI2	4.47E-02	3.08				
Q8WWI1	LMO7	4.48E-02	2.18				
P12268	IMPDH2	4.50E-02	1.81	2.74E-02	1.85		
P04792	HSPB1	4.52E-02	0.56	2.51E-02	0.55	2.03E-02	0.54
Q9GZM8	NDEL1	4.55E-02	2.70				
Q70J99	UNC13D	4.56E-02	1.64				

Q96M89	CCDC138	4.62E-02	3.61				
P10619	CTSA	4.66E-02	4.20	4.99E-02	4.34	1.28E-02	6.65
Q9H4L7	SMARCAD1	4.66E-02	0.26				
Q13501	SQSTM1	4.66E-02	1.73				
Q08J23	NSUN2	4.66E-02	0.88				
Q03164	KMT2A	4.66E-02	0.67				
Q9NQX4	MYO5C	4.66E-02	0.28				
Q15369	ELOC	4.66E-02	4.07				
O75376	NCOR1	4.66E-02	9.14				
Q8IVG5	SAMD9L	4.66E-02	14.88				
Q8TCG1	CIP2A	4.67E-02	0.00				
Q6ZWJ8	KCP	4.67E-02	0.36				
Q9UKX2	MYH2	4.67E-02	0.00				
Q8TEK3	DOT1L	4.75E-02	5.51				
Q99538	LGMN	4.75E-02	0.28	2.75E-02	0.28		
Q13442	PDAP1	4.77E-02	2.39				
Q9Y4Z0	LSM4	4.79E-02	5.41				
Q8WYQ5	DGCR8	4.80E-02	0.02				
Q9BZM2	PLA2G2F	4.80E-02	0.07				
O75914	PAK3	4.81E-02	4.32	2.54E-02	5.28		
P33527	ABCC1	4.82E-02	0.56				
P04062	GBA	4.82E-02	12.72	4.10E-03	22.58		
Q9NQ60	EQTN	4.82E-02	0.14				
Q96E29	MTERF3	4.82E-02	1.97				
Q8NEY1	NAV1	4.83E-02	4.00				
Q93084	ATP2A3	4.83E-02	0.19				
Q13023	AKAP6	4.83E-02	0.05				
Q9Y2V2	CARHSP1	4.83E-02	4.80				
P53794	SLC5A3	4.85E-02	1.21				
Q0VDD8	DNAH14	4.85E-02	3.21				
Q96JY0	MAEL	4.85E-02	5.80				
P48764	SLC9A3	4.85E-02	0.38				
P49619	DGKG	4.85E-02	1.65				
O96011	PEX11B	4.85E-02	1.94	4.68E-03	7.26		
Q9NZ45	CISD1	4.87E-02	3.68	3.61E-02	3.67		
A0FGR9	ESYT3	4.90E-02	5.27				
P09488	GSTM1			4.61E-02	0.13	3.03E-02	0.14
P58107	EPPK1			3.07E-02	0.44	1.07E-02	0.16
O75531	BANF1			3.50E-02	0.18	4.98E-02	0.18
Q92599	SEPTIN8			3.42E-02	0.35	1.94E-02	0.21
P20810	CAST			2.74E-02	0.39	3.42E-02	0.31
Q96N66	MBOAT7			4.18E-02	0.54	3.87E-02	0.47
P15090	FABP4			3.65E-02	0.79	4.25E-02	0.54
P04406	GAPDH			2.94E-02	0.69	8.94E-03	0.65
Q52LJ0	FAM98B					4.24E-02	0.00
P50135	HNMT					4.10E-02	0.06
Q14203	DCTN1					9.81E-03	0.07
P11233	RALA					1.35E-02	0.07
P06737	PYGL					3.33E-02	0.09
P48147	PREP					4.68E-02	0.11
P37059	HSD17B2					3.22E-02	0.19
Q96IJ6	GMPPA					3.66E-02	0.19
P63151	PPP2R2A					1.90E-02	0.20
O95834	EML2					2.24E-02	0.22
Q9H8H3	METTL7A					1.11E-02	0.25
Q12959	DLG1					1.28E-02	0.25
Q04828	AKR1C1					5.53E-03	0.28
P37108	SRP14					5.53E-03	0.29
P31939	ATIC					3.40E-02	0.30
Q13409	DYNC1I2					3.53E-02	0.34
Q9UJU6	DBNL					2.87E-02	0.36

O75436	VPS26A					3.44E-02	0.39
O76070	SNCG					4.46E-02	0.43
Q13510	ASAH1					1.52E-02	0.44
P11766	ADH5					1.18E-02	0.45
O15145	ARPC3					6.92E-03	0.50
P05455	SSB					3.45E-02	0.52
P13010	XRCC5					8.94E-03	0.53
P61158	ACTR3					7.54E-03	0.54
P51991	HNRNPA3					4.63E-02	0.54
Q16629	SRSF7					4.45E-02	0.56
Q5SSJ5	HP1BP3					4.45E-02	0.57
Q01518	CAP1					1.12E-02	0.60
P17858	PFKL					3.63E-02	0.61
P14866	HNRNPL					3.29E-02	0.67
P31943	HNRNPH1					4.87E-02	0.70
P31946	YWHAB					3.97E-02	0.70
P54868	HMGCS2					4.02E-02	0.74
P05386	RPLP1			4.30E-02	0.93		
Q08257	CRYZ			4.30E-02	0.25		
E5RIL1	UPK3BL2			4.99E-02	0.04		
Q9ULW8	PADI3			1.13E-02	0.38		
P52272	HNRNPM			3.79E-02	0.73		
Q13336	SLC14A1			3.44E-02	0.15		
P50895	BCAM			3.76E-02	0.34		
Q4KWH8	PLCH1			4.76E-03	0.10		
P06733	ENO1			3.07E-02	0.58		
O00757	FBP2			4.88E-02	0.51		
O75367	MACROH2A1			4.37E-02	0.69		
O75891	ALDH1L1			2.47E-02	0.21		
P52907	CAPZA1			1.85E-02	0.62		
Q12905	ILF2			4.69E-02	0.45		
Q92817	EVPL			1.36E-02	0.30		
Q00577	PURA			4.88E-02	0.17		
P78371	CCT2			8.99E-04	0.22		
Q8N163	CCAR2			1.85E-02	0.54		
Q92626	PXDN			2.57E-02	0.03		
P14061	HSD17B1			4.38E-02	0.24		
Q7Z406	MYH14			2.75E-02	0.46		
Q9NSI6	BRWD1			8.41E-03	0.00		
O75165	DNAJC13			2.83E-02	0.00		
Q9BVI4	NOC4L			3.15E-02	0.00		
Q9NVM9	INTS13			4.18E-02	0.06		
Q8IXM7	ODF3L1			4.02E-03	0.10		
Q8WTT2	NOC3L			4.61E-02	0.11		
Q9Y2T4	PPP2R2C			2.38E-02	0.12		
Q9Y2J0	RPH3A			1.70E-03	0.13		
P56696	KCNQ4			4.01E-02	0.16		
Q6P3S1	DENND1B			1.08E-03	0.16		
Q5VYS8	TUT7			6.68E-04	0.16		
Q9NZM3	ITSN2			1.79E-02	0.17		
Q9BQ95	ECSIT			4.73E-02	0.18		
P00533	EGFR			1.50E-04	0.18		
A0A087X1G2	TBC1D3K			8.31E-03	0.25		
O43918	AIRE			1.20E-02	0.25		
Q9Y6L6	SLCO1B1			8.61E-03	0.28		
P29597	TYK2			3.47E-02	0.28		
Q9Y4B5	MTCL1			4.31E-04	0.28		
Q9P2P1	NYNRIN			3.89E-02	0.32		
Q09472	EP300			9.71E-03	0.32		
Q7Z5P9	MUC19			2.99E-02	0.33		
P32238	CCKAR			2.15E-02	0.36		

Q5T7M9	DIPK1A			4.12E-02	0.45		
Q9ULR3	PPM1H			2.47E-02	0.14		
Q13885	TUBB2A					1.40E-02	1.42
P07237	P4HB			3.02E-02	1.54	3.24E-02	1.58
P29692	EEF1D					4.02E-02	1.68
P49755	TMED10					3.55E-02	1.70
P13667	PDIA4			3.88E-02	1.97	2.99E-02	1.79
P09382	LGALS1			4.76E-02	1.74	2.76E-02	2.02
P53999	SUB1					4.68E-02	2.13
P54577	YARS1					8.78E-03	2.13
P62888	RPL30					3.10E-02	2.18
P06454	PTMA					3.85E-02	2.22
O94832	MYO1D					2.71E-03	2.53
P04083	ANXA1			4.44E-02	2.54	3.47E-02	2.76
P15531	NME1					2.79E-03	2.81
P80188	LCN2					3.55E-02	3.14
Q6DD88	ATL3			2.02E-02	1.70	4.47E-03	3.16
Q13509	TUBB3					6.92E-03	3.16
Q01581	HMGCS1					1.36E-02	3.20
P34897	SHMT2					1.38E-03	3.28
P53004	BLVRA					3.10E-02	3.63
P35580	MYH10					3.31E-02	3.72
P35659	DEK			8.61E-03	3.99	1.38E-03	3.77
Q06210	GFPT1			4.13E-03	3.07	1.07E-03	3.81
Q96JB5	CDK5RAP3					8.07E-03	3.82
P25774	CTSS					1.35E-02	3.93
Q08379	GOLGA2			3.37E-02	2.94	5.62E-03	3.99
P57088	TMEM33					3.53E-02	4.12
Q9NX40	OCIAD1					1.87E-02	4.31
P11678	EPX			2.24E-04	5.32	9.81E-03	4.32
P21980	TGM2					4.87E-02	4.41
Q14498	RBM39					3.45E-02	4.54
P26885	FKBP2					7.87E-03	4.84
P13611	VCAN			2.99E-02	4.09	8.45E-03	5.16
P08246	ELANE					9.63E-03	5.53
P62993	GRB2					2.33E-02	5.60
Q08945	SSRP1					8.45E-03	6.06
O15355	PPM1G			6.68E-04	10.73	2.59E-02	6.12
P48059	LIMS1					2.91E-02	6.67
P27708	CAD					1.45E-02	6.72
P16070	CD44			2.25E-02	3.18	3.24E-02	6.77
Q9UJS0	SLC25A13					2.79E-03	6.80
Q53GQ0	HSD17B12			4.66E-02	3.11	1.00E-02	6.88
Q13177	PAK2					9.17E-03	6.98
P29373	CRABP2					1.37E-02	7.36
P30419	NMT1					3.10E-02	7.81
Q14739	LBR					3.01E-02	8.55
P29350	PTPN6			8.61E-03	5.58	2.77E-04	8.61
P15586	GNS					1.35E-02	9.14
P17900	GM2A					1.11E-03	11.02
P41252	IARS1					3.57E-02	11.85
Q9Y6E2	BZW2					2.18E-02	13.03
O75886	STAM2					1.66E-03	13.80
O00391	QSOX1			1.87E-02	9.45	1.53E-02	15.50
P84095	RHOG					2.75E-03	16.23
P31689	DNAJA1					1.18E-02	17.02
P23381	WARS1					6.74E-04	17.30
Q9H4M9	EHD1					5.14E-03	35.58
O76094	SRP72					1.61E-02	125.99
P02533	KRT14					2.02E-06	only in MIBC
Q96AY3	FKBP10			1.08E-03	only in MIBC	1.44E-03	only in MIBC

Q9UIV8	SERPINB13					1.44E-03	only in MIBC
Q9Y2Y8	PRG3			8.61E-03	only in MIBC	9.81E-03	only in MIBC
Q70UQ0	IKBIP			8.61E-03	only in MIBC	9.81E-03	only in MIBC
P31151	S100A7					9.81E-03	only in MIBC
P22307	SCP2			3.42E-02	2.88		
P29508	SERPINB3			2.75E-02	6.29		
Q9Y6B6	SAR1B			4.37E-02	4.22		
P31146	CORO1A			3.47E-02	2.67		
Q6ZRV2	FAM83H			5.74E-03	4.05		
P61081	UBE2M			4.69E-02	2.38		
P60866	RPS20			4.69E-02	1.51		
Q15436	SEC23A			4.30E-02	5.04		
P22061	PCMT1			4.79E-02	2.81		
Q9ULV4	CORO1C			1.33E-02	6.59		
Q14019	COTL1			4.30E-02	2.04		
Q8WX93	PALLD			8.61E-03	1.51		
P53007	SLC25A1			3.29E-02	1.90		
P35606	COPB2			4.40E-02	1.66		
P23193	TCEA1			3.01E-03	6.54		
P54727	RAD23B			3.27E-02	1.58		
Q93009	USP7			6.35E-04	19.21		
Q15029	EFTUD2			1.82E-02	7.69		
P60660	MYL6			3.27E-02	1.23		
Q02952	AKAP12			2.22E-02	5.65		
P00492	HPRT1			4.88E-02	2.52		
P53396	ACLY			3.76E-02	3.34		
P46977	STT3A			4.84E-02	1.72		
P61619	SEC61A1			3.48E-02	1.94		
P16949	STMN1			3.74E-02	3.97		
P48444	ARCN1			3.37E-02	1.73		
P09914	IFIT1			2.75E-02	21.26		
Q02388	COL7A1			5.43E-03	7.09		
Q8N2K0	ABHD12			4.99E-02	8.42		
P35914	HMGCL			1.41E-03	4.44		
Q12907	LMAN2			3.96E-02	1.42		
O75844	ZMPSTE24			1.90E-04	7.00		
P62917	RPL8			4.14E-02	1.31		
Q13753	LAMC2			8.61E-03	13.80		
Q13428	TCOF1			2.82E-02	6.81		
P08648	ITGA5			3.29E-02	2.62		
Q9NR28	DIABLO			8.33E-03	4.06		
P27482	CALML3			3.07E-02	1.99		
Q562R1	ACTBL2					4.68E-02	1.54
P22105	TNXB			3.45E-02	1.32		
Q15286	RAB35			3.75E-02	1.36		
O43464	HTRA2			2.89E-02	1.62		
Q9NYF8	BCLAF1			9.20E-03	1.98		
Q14162	SCARF1			3.54E-02	2.16		
O94776	MTA2			3.35E-02	2.53		
P35228	NOS2			1.54E-02	2.75		
Q7Z4V5	HDGFL2			2.75E-02	2.82		
Q9UPA5	BSN			1.27E-02	2.97		
Q8IZ81	ELMOD2			4.76E-02	3.13		
O00329	PIK3CD			1.53E-02	3.33		
Q9UFD9	RIMBP3			1.82E-02	4.10		
P53680	AP2S1			3.52E-02	4.26		
O14561	NDUFAB1			3.79E-02	4.36		
Q63HR2	TNS2			3.29E-02	5.07		
Q9ULD0	OGDHL			1.85E-02	5.46		
Q13796	SHROOM2			2.82E-02	5.51		
P08579	SNRPB2			3.02E-02	5.56		

Q16799	RTN1			4.48E-02	5.69				
Q9UBC5	MYO1A			5.70E-03	5.71				
Q9NTZ6	RBM12			5.55E-03	6.00				
Q96MX6	WDR92			8.61E-03	6.96				
O43747	AP1G1			6.31E-04	7.42				
Q5T8P6	RBM26			2.99E-02	8.03				
Q9UL68	MYT1L			2.68E-02	8.44				
O75400	PRPF40A			2.90E-03	9.09				
Q04206	RELA			6.21E-03	9.29				
Q96RS6	NUDCD1			1.27E-02	9.40				
Q9BXW7	HDHD5			1.22E-04	9.95				
Q5T4S7	UBR4			5.66E-03	10.74				
P21108	PRPS1L1			1.27E-02	11.31				
P30566	ADSL			8.99E-04	12.60				
Q9UQN3	CHMP2B			8.61E-03	18.71				
Q6PIF6	MYO7B			2.68E-02	19.74				
Q99442	SEC62			8.61E-03	24.52				
Q9HAK2	EBF2			5.35E-08	30.11				
O60293	ZFC3H1			1.60E-04	59.45				
Q6WCQ1	MPRIP			1.08E-03	only in MIBC				
Q14008	CKAP5			1.08E-03	only in MIBC				
O75592	MYCBP2			1.08E-03	only in MIBC				
P82094	TMF1			8.61E-03	only in MIBC				
A2RUR9	CCDC144A			8.61E-03	only in MIBC				
Q13637	RAB32			8.61E-03	only in MIBC				
Q9UHN6	CEMIP2			8.61E-03	only in MIBC				
P22694	PRKACB					5.53E-03	6.16		
Q5VZ66	JAKMIP3			2.22E-02	0.00				
Q96LZ2	MAGEB10			3.50E-02	0.00				
O00622	CCN1			4.47E-02	0.04				
O75461	E2F6			3.81E-04	0.17				
P19623	SRM			3.44E-02	0.18				
Q96EY9	ADAT3			2.87E-03	0.21				
O95407	TNFRSF6B			3.65E-03	0.29				
Q96PL5	ERMAP			1.36E-02	0.41				
O00499	BIN1			3.47E-02	4.87				
Q9H8J5	MANSC1			4.38E-02	5.63				
P34913	EPHX2			2.94E-02	6.26				
P29992	GNA11			1.50E-04	8.02				
Q6NUI2	GPAT2			2.00E-03	8.06				
Q8N9V3	WDSUB1			4.51E-03	3.83				
Q9P0U1	TOMM7			1.27E-02	5.46				
