# Supplementary material for: Repurposing Amiodarone for Bladder Cancer Treatment
Source: Cancer Res Commun. 2025 Jun 4;5(6):906–20. doi: 10.1158/2767-9764.CRC-24-0433 (PMC12134865; doi:10.1158/2767-9764.CRC-24-0433)
Supplement: Supplementary File 1 — The analysis based on the previously generated proteomics data [file crc-24-0433_supplementary_file_1_suppsf1.docx]

**Supplementary File**

The analysis was based on the previously generated proteomics data (1). One hundred and twelve RAW data files (NMIBC: n=58 pTa, n=38 pT1, MIBC: n=16 pT2+) generated from the liquid chromatography coupled with tandem mass spectrometry (LC-MS/MS) Orbitrap LTQ Velos platform were analyzed using two different software, namely MaxQuant v 1.6.7.0 (Andromeda search engine) and the Proteome Discoverer 2.4 (SEQUEST search engine). In both cases, database searches were carried out against *Homo Sapiens (reviewed)* Swiss-Prot Database containing the canonical sequences only. Carbamidomethylation of cysteine was considered as static modification, while oxidation of methionine and proline were set as dynamic modifications. A precursor mass tolerance of 5 ppm and fragment mass tolerance of 0.05 Da were considered, together with two missed cleavage sites. Data obtained using the Proteome Discoverer were further processed with a clustering approach, as previously described (2). This includes data exporting at the peptide level and calibration of the retention time over a reference dataset. Three different analytical approaches were followed [(Proteome Discoverer – identification of peptides based on the target False discovery rate (FDR) of 1%, and without FDR threshold) and MaxQuant (1% FDR)]. FDR validation was conducted using Percolator node. Part per million (ppm) normalization was applied based on the following formula: normalized peak area = (peptide peak area/total peak area in a sample) × 1,000,000. The proteins quantified from each of the aforementioned processing strategies were subject to separate statistical analysis (MIBC vs NMIBC) using Mann Whitney test (wilcox.test function in R), followed by Benjamini Hochberg (BH) corrections (p.adjust function in R). Differentially abundant proteins (DAPs) were defined as significant in at least one approach (BH adjusted p < 0.05) and detected in more than 10% of samples (in at least one analysis) and showed an agreement in fold change directionality among significant findings.

Transcriptomics meta-analysis data were retrieved from the previously published work (3). 495 patients with NMIBC (n=229 pTa, n=262 pT1, n=4 pTis), and 563 patients with MIBC (n=371 pT2, n=146 pT3, and n=46 pT4) were included. Differential expression analysis (MIBC vs NMIBC) was conducted using Mann-Whitney test, followed by the adjustment for multiple using BH method. An AUC-based discriminatory analysis was conducted between MIBC versus NMIBC. The AUC calculation was performed using R core software as well as the contributed cran-package ROCR. Those differentially expressed genes (DEG) with AUC ≥ 0.6 and being significant after adjustment for multiple testing were considered for further downstream analysis.

Features from BcCluster (4) were filtered based on the following criteria: i) originating from tissue samples (blood and urine samples were excluded), ii) p-value ≤ 0.05 and iii) with regulation values of “up” or “down” (other values omitted).

The DAPs, DEGs, and features from BcCluster were compiled in a BC molecular signature by initially inspecting the consistency of directionality (i.e., up/down regulation) of each feature among these different datasets. Features that demonstrated inconsistency in directionality across different datasets were excluded. Subsequently, the functional relationships between the disease phenotype and known drug compounds were analyzed using the Connectivity Map Tool (CMap) (5). The BC signature was utilized to query known drug signatures representing the response of human cancer cells to treatment. Given the limited number of features that can be used as input for the CMap tool, ten randomized lists of 500 features each were generated. Only those drugs/compounds that exhibited significant negative enrichment (p-value < 0.05) in at least one CMap analysis were considered for further examination.

To select the most promising drug candidates, the compounds underwent thorough annotation using information available in various web-based tools, public databases, and the literature. For this purpose, the OpenTargets platform (6) was utilized to gather data on completed or ongoing clinical trials and studies related to BC or other cancers. The availability of the compounds was assessed by searching the PubChem database (7). Additionally, the aspect of novelty concerning BC was explored. Literature searches were conducted to find scientific evidence for the predicted compounds/active ingredients' use: (i) in the context of BC and (ii) in the context of cancer in general. The drugs' status as essential medicine (8) and their FDA approval status (<https://www.accessdata.fda.gov/scripts/cder/daf/index.cfm>) were also investigated. Furthermore, the drugs' indications and drug classes were checked in the MeSH database (MeSH https://meshb.nlm.nih.gov/) and in KEGG (9). The Drug-repurposing Hub (https://clue.io/repurposing-app), a freely accessible repository, was utilized to gain insights into the potential mechanisms of action. The primary focus was on compounds belonging to the 100 most important compounds, prioritized based on their average enrichment score.

REFERENCES

1. Stroggilos R, Mokou M, Latosinska A, Makridakis M, Lygirou V, Mavrogeorgis E, et al. Proteome-based classification of Nonmuscle Invasive Bladder Cancer. Int J Cancer. 2020;146(1):281-94.

2. Lygirou V, Latosinska A, Makridakis M, Mullen W, Delles C, Schanstra JP, et al. Plasma proteomic analysis reveals altered protein abundances in cardiovascular disease. J Transl Med. 2018;16(1):104.

3. Stroggilos R, Frantzi M, Zoidakis J, Mokou M, Moulavasilis N, Mavrogeorgis E, et al. Gene Expression Monotonicity across Bladder Cancer Stages Informs on the Molecular Pathogenesis and Identifies a Prognostic Eight-Gene Signature. Cancers (Basel). 2022;14(10).

4. Bhat A, Mokou M, Zoidakis J, Jankowski V, Vlahou A, Mischak H. BcCluster: A Bladder Cancer Database at the Molecular Level. Bladder Cancer. 2016;2(1):65-76.

5. Lamb J, Crawford ED, Peck D, Modell JW, Blat IC, Wrobel MJ, et al. The Connectivity Map: using gene-expression signatures to connect small molecules, genes, and disease. Science. 2006;313(5795):1929-35.

6. Ochoa D, Hercules A, Carmona M, Suveges D, Gonzalez-Uriarte A, Malangone C, et al. Open Targets Platform: supporting systematic drug-target identification and prioritisation. Nucleic Acids Res. 2021;49(D1):D1302-d10.

7. Kim S, Chen J, Cheng T, Gindulyte A, He J, He S, et al. PubChem 2023 update. Nucleic Acids Res. 2023;51(D1):D1373-d80.

8. Medicines E. WHO Model List of Essential Medicines—23rd List; 2023. 2023.

9. Kanehisa M, Furumichi M, Sato Y, Kawashima M, Ishiguro-Watanabe M. KEGG for taxonomy-based analysis of pathways and genomes. Nucleic Acids Res. 2023;51(D1):D587-d92.
